# NucDiff: in-depth characterization and annotation of differences between two sets of DNA sequences

**DOI:** 10.1186/s12859-017-1748-z

**Published:** 2017-07-12

**Authors:** Ksenia Khelik, Karin Lagesen, Geir Kjetil Sandve, Torbjørn Rognes, Alexander Johan Nederbragt

**Affiliations:** 1 Biomedical Informatics Research Group, Department of Informatics, University of Oslo, PO Box 1080, 0316 Oslo, Norway; 2 Norwegian Veterinary Institute, PO Box 750 Sentrum, 0106 Oslo, Norway; 3 Department of Microbiology, Oslo University Hospital, Rikshospitalet, PO Box 4950 Nydalen, 0424 Oslo, Norway; 4 Centre for Ecological and Evolutionary Synthesis, Department of Biosciences, University of Oslo, PO Box 1066 Blindern, 0316 Oslo, Norway

**Keywords:** Whole-genome alignment, Comparative analysis, Whole-genome assembly, Annotation of differences

## Abstract

**Background:**

Comparing sets of sequences is a situation frequently encountered in bioinformatics, examples being comparing an assembly to a reference genome, or two genomes to each other. The purpose of the comparison is usually to find where the two sets differ, e.g. to find where a subsequence is repeated or deleted, or where insertions have been introduced. Such comparisons can be done using whole-genome alignments. Several tools for making such alignments exist, but none of them 1) provides detailed information about the types and locations of all differences between the two sets of sequences, 2) enables visualisation of alignment results at different levels of detail, and 3) carefully takes genomic repeats into consideration.

**Results:**

We here present NucDiff, a tool aimed at locating and categorizing differences between two sets of closely related DNA sequences. NucDiff is able to deal with very fragmented genomes, repeated sequences, and various local differences and structural rearrangements. NucDiff determines differences by a rigorous analysis of alignment results obtained by the NUCmer, delta-filter and show-snps programs in the MUMmer sequence alignment package. All differences found are categorized according to a carefully defined classification scheme covering all possible differences between two sequences. Information about the differences is made available as GFF3 files, thus enabling visualisation using genome browsers as well as usage of the results as a component in an analysis pipeline. NucDiff was tested with varying parameters for the alignment step and compared with existing alternatives, called QUAST and dnadiff.

**Conclusions:**

We have developed a whole genome alignment difference classification scheme together with the program NucDiff for finding such differences. The proposed classification scheme is comprehensive and can be used by other tools. NucDiff performs comparably to QUAST and dnadiff but gives much more detailed results that can easily be visualized. NucDiff is freely available on https://github.com/uio-cels/NucDiff under the MPL license.

**Electronic supplementary material:**

The online version of this article (doi:10.1186/s12859-017-1748-z) contains supplementary material, which is available to authorized users.

## Background

Advances in whole genome sequencing strategies and assembly approaches have brought on a need for methods for comparing sets of sequences to each other. Common questions asked are how assemblies of the same read set obtained with different assembly programs differ from each other, or how genomes from different strains of the same bacterial species differ from each other. Whole genome alignment (WGA) methods are often used for performing such analyses and have long been studied in bioinformatics. WGA “is, in general, the prediction of homologous pairs of positions between two or more sequences” [1]. WGA is mainly used for identifying conserved sequences between genomes, e.g. genes, regulatory regions, non-coding RNA sequences, and other functional elements [2, 3], thus aiding, for instance, genome (functional) annotation, detecting large scale evolutionary changes between genomes, and phylogenetic inference [1, 2]. This field has been under continuous development since the 1970s, and many methods and tools for WGA have been created. Reviews of existing methods and tools can be found in [1, 4, 5].

For the purpose of detecting differences between sequence sets, tools that can be used to perform WGA analysis should come with certain features. First, they should be able to deal with very fragmented genomes, structural rearrangements, genome sequence duplications, and various differences that are often related to repeated regions. Second, the comparative analysis results should provide information about the types of differences and their locations. This information should be stored in ways suitable for further analysis. Such comparison information may, for example, be used for scaffolding purposes, for reference-assisted genome assembly, assembly error detection, and comparison of different assemblies. Third, they should enable visualisations of alignment results at different levels of detail. Global scale visualisation can be used for examining duplications, structural rearrangements, and uncovered regions, while local scale visualisation can provide information about small differences, such as substitutions, insertions and deletions (collectively called ‘indels’).

Three different tools are available today that partially satisfy these criteria: MAUVE [6], QUAST [7] and dnadiff [8]. MAUVE performs multiple genome alignment, identifies conserved genomic regions, rearrangements and inversions in these regions, and the exact sequence breakpoints of such rearrangements across multiple genomes as well as nucleotide substitutions and small indels [6]. It also enables analysis of results through interactive visualisation and stores information in separate files. However, only information about small differences (substitutions, indels) is easily accessible without running accessory programs.

QUAST is a tool for quality assessment of genome assemblies, which outputs different metrics on assembly quality in the presence of a reference genome. It gives information about the locations of structural and long local differences, specifying the types of structural differences only. QUAST enables visualisation in an accompanying genome browser called Icarus. However, QUAST lacks visualisation of small local differences, only providing summary statistics for them.

Dnadiff is a wrapper for the NUCmer alignment program from MUMmer [9] that quantifies the differences and provides alignment statistics and other high-level metrics [8]. Similar to QUAST, dnadiff can be used for quality assessment of assemblies and comparison of genomes, but it does not provide any visualization of the detected differences.

Here we present the tool NucDiff, which uses the NUCmer, delta-filter and show-snps programs from MUMmer for sequence comparison. NUCmer aligns sequences and outputs information about aligned sequence regions. Rigorous analysis of the relative positions of these regions enables detection of various types of differences, including rearrangements and inversions, and in some cases also to ascertain their connection with repeated regions. NucDiff identifies the differences between two sets of closely related sequences and classifies the differences into several subtypes. The precise locations of all differences using coordinates systems with respect to both input sequences are output as GFF3 (Generic Feature Format version 3, [10]) files. These precise locations enables both visualisation and further analysis. The information provided by NucDiff can thus significantly help clarify how two sets of sequences differ.

## Implementation

NucDiff determines the various types of differences between two sets of sequences, usually referred to as a reference genome and a query, by parsing alignment results produced by the NUCmer, delta-filter and show-snps programs from the MUMmer sequence alignment package [9]. NUCmer performs DNA sequence alignment, while delta-filter filters the alignment results according to specified criteria. With the settings used by NucDiff by default, delta-filter also selects the longest consistent alignments for the query sequences. NUCmer alignment results contain information about fragments of sequences that match, which we here refer to as query and reference fragments. NUCmer output contains the exact coordinates of all fragments in relation to their source sequences, directions of query fragments relative to corresponding reference fragments, and percent similarity of the alignment. The show-snps results contain information about all inserted, deleted and substituted bases in the query fragments compared to the corresponding reference fragments.

**Fig. 1:**
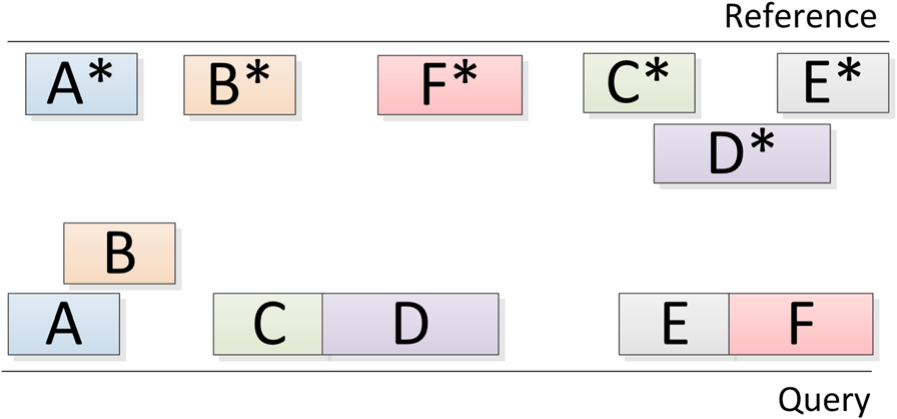
NUCmer alignment. A,...,F represent query fragments, while A*,.., F* represent reference fragments. A*-A, …, F*-F are matches according to NUCmer

If we represent the output fragments as blocks on the query and reference sequences, then a possible NUCmer alignment result may look as illustrated in Fig. 1.

During the alignment process, NUCmer searches for maximal exact matches of a given minimum length, then clusters these matches to form larger inexact alignment regions, and finally extends alignments outwards from each of the matches to join the clusters into a single high scoring pairwise alignment [11]. If the query sequences contain long (by default, more than 200 bp) insertions, deletions, substitutions, or any structural rearrangements, the alignment will be broken and subsequently consist of separate fragments with the ends coinciding with the locations of these differences. NucDiff classifies the alignment fragments by analysing the placement of all pairs of neighbouring query fragments (A-B, B-C, etc. in Fig. 1), their placement on the reference sequences (A*-B*, B*-C*, etc. in Fig. 1), and their orientations (5′ to 3′, or 3′ to 5′). The obtained differences together with the differences from show-snps form the set of all differences between query and reference sequences.

**Fig. 2:**
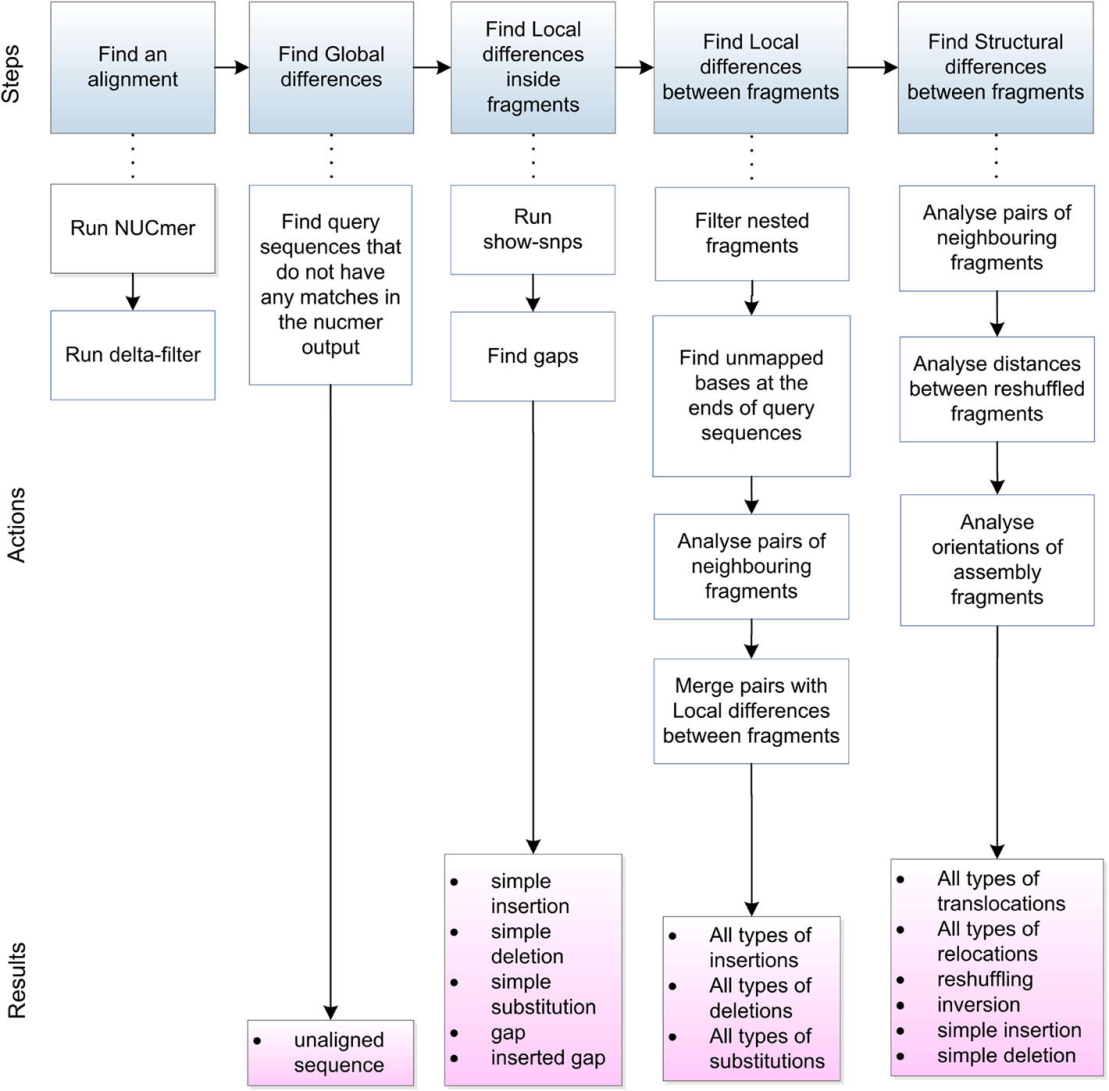
NucDiff workflow. The top blue boxes correspond to the NucDiff steps described in the Stepwise detection of differences section. The white boxes under each step represent the main actions performed during this step. The lower pink boxes give information about types of differences that are detected at each step

The NucDiff workflow is shown in Fig. 2. An overview of all types of differences that NucDiff is able to detect is presented in the Types of differences section. A description of the steps involved in their detection is given in the Stepwise detection of differences section.

### Types of differences

**Fig. 3:**
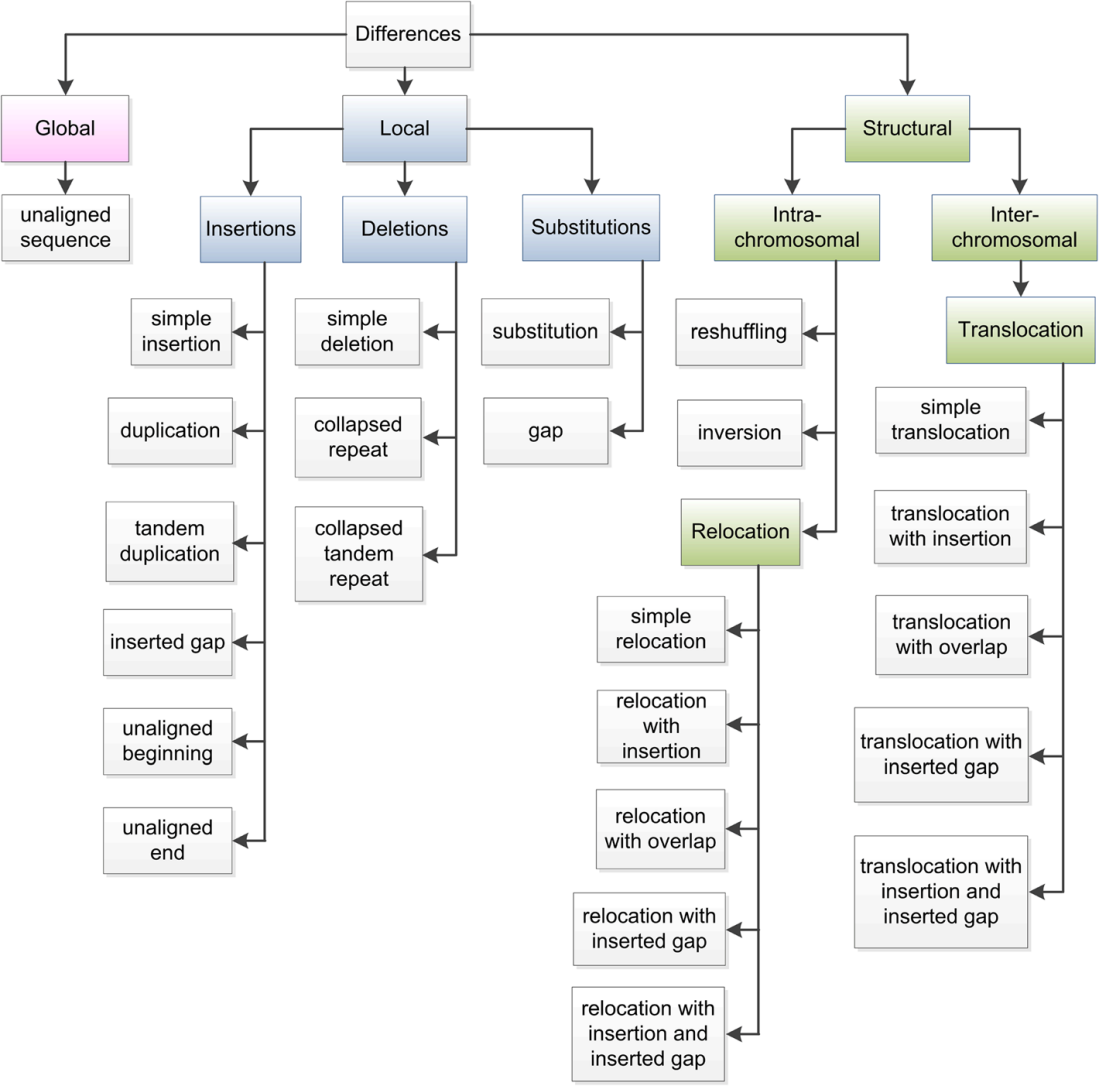
Classification of the types of differences. Group names are given in coloured boxes with capitalised names and the specific types are given in white boxes and with lowercase names

We classify all types of differences into 3 main groups: global, local and structural (Fig. 3). These differences are here denoted as changes in the query when compared to the reference.

#### Global differences

Global differences affect the whole query sequence. This group consists of only one type, called unaligned sequence.

- unaligned sequence - a query sequence that has no matches of length equal to or longer than a given number of bases (65 by default) with the reference genome.

#### Local differences

Local differences involve various types of insertions, deletions and substitutions. NucDiff distinguishes between six types of insertions (the insertion subgroup in Fig. 3):

- simple insertion - an insertion of bases in the query sequence that were not present anywhere on the reference genome.
- duplication - an insertion in the query sequence of an extra copy of some reference sequence not adjacent to this region, creating an interspersed repeat, or increasing the copy number of an interspersed repeat
- tandem duplication - an insertion of an extra copy of some reference sequence region adjacent to this region in the query sequence
- inserted gap - an insertion of unknown bases (N’s) in the query sequence in a region which is continuous (without a gap) in the reference, or which results in an elongation of a region of unknown bases in the reference.
- unaligned beginning - unaligned bases in the beginning of a query sequence
- unaligned end - unaligned bases at the end of query sequence

There are several types of deletions (the deletion subgroup in Fig. 3):

- simple deletion - a deletion of some bases, present in the reference sequence, from a query sequence
- collapsed repeat - a deletion of one copy of an interspersed repeat from the reference sequence in a query sequence
- collapsed tandem repeat - a deletion of one or more tandem repeat units from the reference sequence in a query sequence

And, last, there are two types of substitutions (the substitution subgroup in Fig. 3):

- substitution - a substitution of some reference sequence region with another sequence of the exact same length not present anywhere in the reference genome (note that this sequence is not categorised as unaligned sequence because it is within a fragment that overlaps between query and reference). SNPs can be considered as a subcategory of substitutions.
- gap - a substitution where a reference subsequence is replaced by an unknown sequence (N’s) of the same length. If the query has an enlarged gap, it will be classified as a combination of a gap and an inserted gap, while a shortened gap is classified as a gap and a simple deletion.

#### Structural differences

NucDiff detects several structural differences. These can be grouped into intra- and inter-chromosomal differences, and some of these contain groups of types:

- translocation - a group of different types of inter-chromosomal structural rearrangements which occur when two regions located on different reference sequences are placed nearby in the same query sequence. The detailed description of all translocation types is given in the Structural difference detection between aligned fragments section.
- relocation - a group of different types of intra-chromosomal structural rearrangements which occur when two regions located in different parts of the same reference sequence are placed nearby in the same query sequence. The detailed description of all relocation types is given in the Structural difference detection between aligned fragments section.
- reshuffling - an intra-chromosomal structural rearrangement which occurs when several neighbouring reference sequence regions are placed in a different order in a query sequence.
- inversion - an intra-chromosomal structural rearrangement which occurs when a query sequence region is the reverse complement of a reference sequence region.

**Fig. 4:**
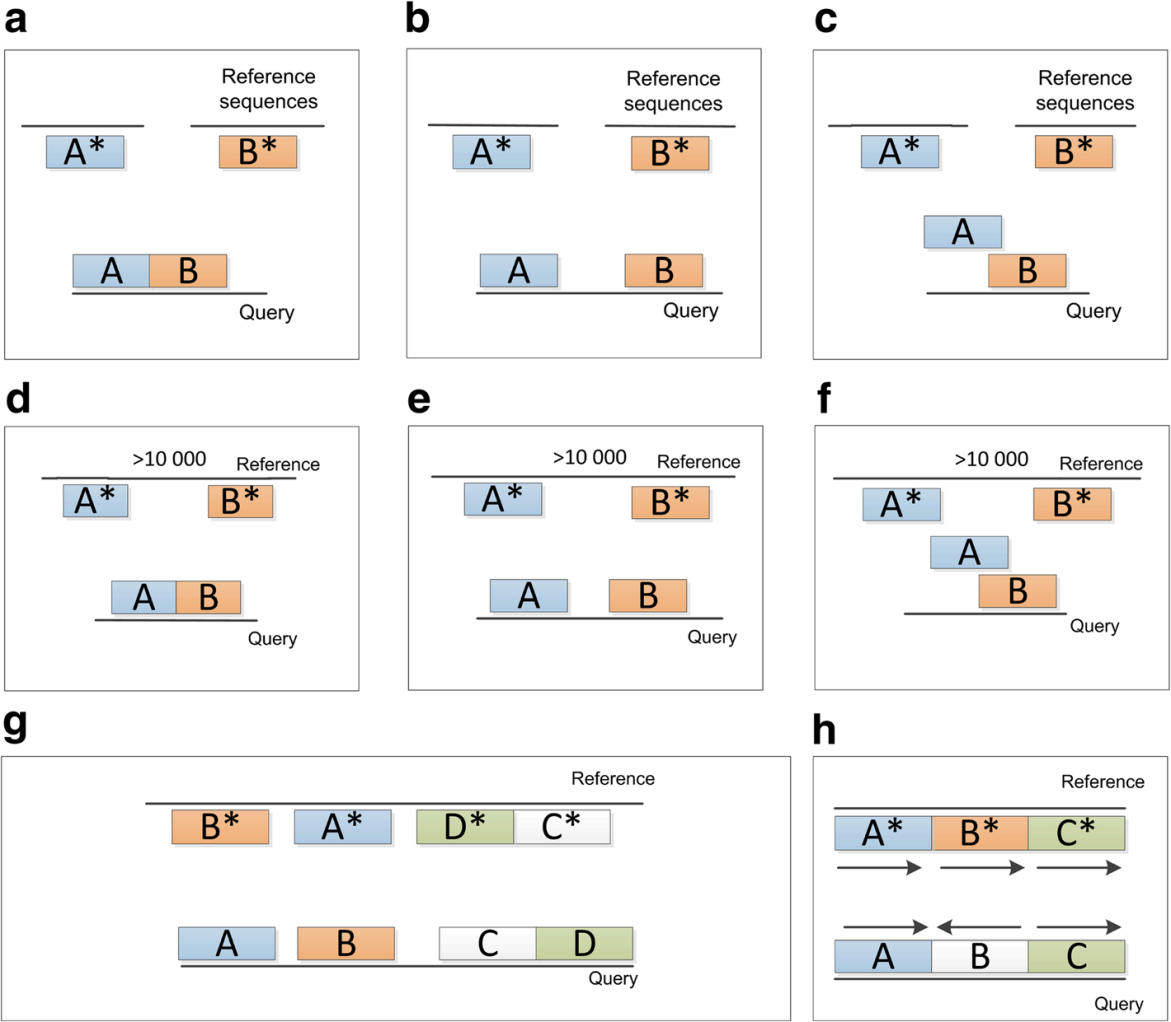
Examples of structural differences. **a** Simple translocation. **b** Translocation with insertion/with inserted gap/with insertion and inserted gap. **c** Translocation with overlap. **d** Simple relocation. **e** Translocation with insertion/with inserted gap/with insertion and inserted gap. **f** Relocation with overlap. **g** Reshuffling. **h** Inversion

The translocation type belongs to the inter-chromosomal subgroup, while relocation, reshuffling and inversion types belong to the intra-chromosomal subgroup (see Fig. 3). Examples of structural differences are given in Fig. 4.

### Stepwise detection of differences

The steps in this section refer to Fig. 2.

#### Global difference detection

NucDiff starts the detection of differences by finding unaligned sequence differences. NUCmer does not output any information about sequences without mapped subsequences longer or equal to a predefined length. Therefore, to find unaligned sequences, NucDiff looks for query sequences with names not mentioned in the NUCmer output. By default, all query sequences shorter than 65 bp will be treated as unaligned sequences. This threshold may be changed using the NUCmer minimum cluster length option.

#### Local difference detection inside aligned fragments

Four types of simple differences may be detected inside the query fragments: simple insertion, simple deletion, simple substitution and gap. The lengths of the differences of these types are limited by how far NUCmer will attempt to extend poorly scoring regions before giving up and are up to 200 bases by default (this threshold may be changed using the NUCmer minimum length of a maximal exact match parameter). Information about the positions of all local differences, except gaps, is found in the show-snps output file. NucDiff parses this file to find simple insertions, simple deletions, and substitutions. To find gaps, NucDiff searches for N’s in the query fragment sequences and outputs their locations.

#### Local difference detection between aligned fragments

NucDiff starts with examining the reason for alignment fragmentation by looking at fragmentation caused by local differences. First, it filters nested fragments in the query and reference sequences. A query nested fragment occurs when two (nearly) identical reference sequence regions have been merged together into one fragment in the query sequence. A reference nested fragment occurs when one reference sequence region is duplicated in the query sequence. Nested fragments provide important information about duplications and collapsed repeats. However, they can cause rather complicated interactions between aligned fragments, which can be difficult to resolve programmatically. Thus, the nested fragments are discarded, and all duplications and collapsed repeats are detected as simple insertions and deletions at later stages of the analysis. Then, NucDiff identifies bases in both ends of the query sequences that were not mapped to the reference sequences. Such bases will be output as unaligned beginning and unaligned end differences.

NucDiff next searches for pairs of neighbouring fragments that were not joined together by NUCmer during the alignment process due to the presence of simple differences, rather than structural differences. Such pairs of fragments should satisfy the following criteria:

- The pair of query fragments as well as the corresponding pair of reference fragments may overlap, be adjacent to each other, or be separated by an inserted region not mapped anywhere on the reference genome.
- The two query fragments should have the same direction. Their two corresponding reference fragments should also have the same direction, but it may be opposite to the direction of the query fragments.
- If the query fragments have the same direction as their corresponding reference fragments, then the reference fragments should be placed in the same order as the query fragments ([Additional file 1: Figure S1a]).
- If the query fragments have the reverse direction of their corresponding reference fragments, then the reference fragments should be in reverse order ([Additional file 1: Figure S1a]).
- The distance between corresponding reference fragments should not be more than a user-defined distance, by default 10,000 bases.

If all these criteria are fulfilled, NucDiff determines the differences based on the placement of the query and reference fragments relative to each other. Examples of all possible placement cases and the corresponding differences are shown in [Additional file 1: Table S1].

After detecting differences between the current pair of neighbouring fragments, NucDiff merges the pair of reference fragments as well as the pair of query fragments together, creating new continuous reference and query fragments, and then searches for the next pair.

#### Structural difference detection between aligned fragments

Fragments not merged during the previous step were kept separate by NUCmer due to structural rearrangements between the query and reference sequences. First, NucDiff searches for translocations, which is one type of inter-chromosomal differences, by searching for a pair of neighbouring query fragments that correspond to fragments located on different reference sequences. We distinguish between 5 types of translocations depending on the placement of the query fragments relative to each other (see also examples in Fig. 4a-c):

- simple translocation - a translocation where two query fragments are placed adjacent to each other.
- translocation with insertion - a translocation where two query fragments have a stretch of bases (not N’s) inserted between them, not mapped anywhere on the reference genome. The inserted region is treated as a simple insertion difference.
- translocation with inserted gap - a translocation where two query fragments have a stretch of unknown bases (N’s) inserted between them. The inserted region is treated as an inserted gap difference.
- translocation with insertion and inserted gap - a translocation where two query fragments have a stretch of bases (A, C, G, T or N’s) inserted between them, not mapped anywhere on the reference genome. The inserted region is treated as both a simple insertion and an inserted gap.
- translocation with overlap - a translocation with a partial overlap between the two query fragments.

In the next step, NucDiff searches for relocations, which is one type of intra-chromosomal differences, by looking for pairs of neighbouring query fragments that were mapped to fragments located on the same reference sequence (e.g. the same chromosome) but separated from each other by at least 10,000 bases, by default. In addition, these fragments should not belong to the group of query fragments placed nearby each other (with the distance between each pair less than 10,000 bases) on the reference sequence in the wrong order, as that would be considered as a reshuffling (see further down). If these two conditions are fulfilled, then there is a relocation. There are 5 types of relocations (see also examples in Fig. 4d-f):

- simple relocation - a relocation where two query fragments are placed adjacent to each other.
- relocation with insertion - a relocation where two query fragments have a stretch of bases (not N’s) inserted between them, not mapped anywhere on the reference genome. The inserted region is treated as a simple insertion difference.
- relocation with inserted gap - a relocation where two query fragments have a stretch of unknown bases (N’s) inserted between them. The inserted region is treated as an inserted gap difference.
- relocation with insertion and inserted gap - a relocation where two query fragments have a stretch of bases (both ATGC’s and N’s) inserted between them, not mapped anywhere on the reference genome. The inserted region is treated as both a simple insertion and an inserted gap.
- relocation with overlap - a relocation with a partial overlap between the two query fragments.

For circular genomes, there is one special case that causes alignment fragmentation: when the start of the query sequence does not coincide with the start of the reference sequence ([Additional file 1: Figure S2]). It satisfies all the criteria for relocations but is not treated as a difference, although it is included in the output.

In the case of translocations and relocations, the query and the corresponding reference fragments may be placed in any direction and order relative to each other. The translocated fragment may contain none, two or more relocated fragments inside. Before the detection of the types of relocations and translocations, NucDiff searches for the pairs of relocated or translocated query fragments that have an overlap between corresponding reference fragments. If such a pair is found, NucDiff truncates the rightmost fragment, so the overlap disappears. In this case information about the repeated nature of the insertion events will be lost.

Third, NucDiff searches for a group of nearby query fragments whose corresponding reference fragments are located on the same reference sequence (chromosome) but in a different order. The distance between two neighbouring reference fragments should not be more than 10,000 bases. If a group satisfying these conditions is found, then there is a reshuffling difference in the query. There may be simple insertion and simple deletion differences between reshuffled fragments. To find them, NucDiff first truncates fragments so that all overlaps between query or reference fragments are removed. It then searches for unmapped bases between neighbouring query fragments to find simple insertions and then searches for unmapped bases between neighbouring reference fragments to find simple deletions.

Finally, NucDiff searches for the last type of intra-chromosomal structural difference, inversions. If a query sequence has several mapped fragments and one or more of them, but not all, have directions opposite to the directions of the corresponding reference fragments, then such fragments are inversions. Some examples of possible alignments of query sequences in cases with reshuffling and inversion are shown in Fig. 4g-h.

Reshufflings and inversions may be present inside translocated and relocated fragments. During reshuffling detection, the directions of reshuffled fragments are not taken into account. Their directions are checked during the inversion detection step. Simple insertions and simple deletions found during this step may be connected to repeated regions, but this connection will not be detected.

### Datasets

We created ten simulated reference and query DNA sequences. The genomes were constructed from random DNA sequences, and different types of controlled genome modifications were subsequently applied to these sequences (e.g. relocation of different fragments, or deletions, or duplications of fragments). The detailed description of implemented genome modifications can be found in [Additional file 1: Table S2].

In addition, we used data produced for the GAGE-B article [12] for the demonstrations of the ﻿comparison of several assemblies. The assemblies from the ABySS [13], CABOG [14], MaSuRCA [15], SGA [16], SOAPdenovo [17] (shown as SOAP in the figures), SPAdes [18] and Velvet [19] assemblers for *Vibrio cholerae* based on HiSeq reads were used. These assemblies together with the *V. cholerae* reference genome were downloaded from the GAGE-B website [20].

For the demonstration of the comparison of genomes from different strains of the same species, 22 *Escherichia coli* K12 reference genomes were downloaded from the NCBI database [21]. Their accession numbers can be found in [Additional file 1: Table S3]. In the sections with the demonstrations, we also used annotations for the *V. cholerae* reference genome and *E. coli* K12 MG1655. They were downloaded from the NCBI database [22, 23], respectively.

## Results

### The NucDiff tool

We have created a tool, called NucDiff, which is primarily aimed at locating and categorizing differences between any two sets of closely related nucleotide sequences. It is able to handle very fragmented genomes and various structural rearrangements. These features make NucDiff suitable for comparing, for instance, different assemblies with each other, or an assembly with a reference genome. NucDiff first runs the NUCmer, delta-filter and show-snps programs from MUMmer and parses the alignment results to detect differences. These differences are subsequently categorized according to a carefully defined classification scheme of all possible differences between two sequences.

A unique feature of NucDiff is that it provides detailed information about the exact genomic locations of the differences in the form of four GFF3 files: two files with information for small and medium local differences that do not cause alignment fragmentation, two others for structural differences and local differences that cause alignment fragmentation. All locations of the differences are output in query - and reference-based coordinates, separately. Each GFF3 entry is additionally annotated with the location of the difference in the opposite coordinate system as well. A detailed description of the format of these GFF files can be found in the GitHub repository of NucDiff. NucDiff also finds the coordinates of mapped blocks (the query sequences split at the points of translocation, relocation, inversions, and/or reshuffling) and then stores them in the GFF3 files, one based on query coordinates and another with reference-based coordinates. Uploading these GFF3 files into a genome browser such as the Integrated Genome Viewer (IGV) [24, 25] enables visualisation of the differences as well as the coverage of a reference genome by query sequences, making it possible to see all uncovered reference bases or if any reference regions are covered multiple times.

In addition, NucDiff generates a summary file containing information about the number of differences of each type. The detailed level of reporting enables users to create their own custom summary from the NucDiff output (e.g. taking into account the length of differences, joining several types of differences together, and so on) if desired.

### Effect of different MUMmer parameters

The alignment results parsed by NucDiff depend on the values of the input parameters for two MUMmer programs, NUCmer and delta-filter. NUCmer performs DNA sequence alignment, while delta-filter filters the alignment results according to specified criteria. Running these programs with different input parameters may result in alternative sets of matches, since the choice of parameters affects the sensitivity of the detection of matching sequence fragments as well as the stringency of the subsequent filtering. To analyse the influence of the different parameters on the alignment and on the subsequent NucDiff results, we compared the results of running NucDiff on the simulated genomes described in the Datasets section with different NUCmer and delta-filter input parameters values. The specific values for each test can be found in [Additional file 1: Table S4]. We also ran one test to enable comparison of QUAST and NucDiff as described in Comparison with QUAST section, since QUAST uses the same underlying tools as NucDiff.

The locations and types of simulated differences were compared with the results obtained from NucDiff, and the number of correctly detected differences was calculated for each test (see [Additional file 1] for details). The results with the total average number of correctly detected expected differences for each type are presented in Table 1. The detailed results for each implemented modification case (see in [Additional file 1: Table S2]) and for each parameter configuration set can be found in [Additional file 2].

**Table 1:** Average number of correctly detected simulated differences by NucDiff with different parameter settings and QUAST

Difference	Truth	Default	c30	c120	l10	l65	b80	b350	QUAST-like	QUAST
insertion	1650	1634	1634	1634	1634	1634	1632	1634	1634	858
deletion	1719	1678	1678	1678	1677	1678	1679	1676	1674	465
duplication	251	136	122	150	136	136	137	124	136	196
tandem_duplication	60	57	57	57	57	57	60	54	57	57
collapsed_repeat	58	53	53	53	53	53	54	51	53	53
collapsed_tandem_repeat	59	55	55	55	55	55	56	53	55	55
relocation	217	127	142	108	127	127	136	112	127	130
relocation-insertion	13	13	13	13	13	13	13	13	13	13
relocation-insertion_ATGC	13	13	13	13	13	13	13	13	13	13
relocation-inserted_gap	13	13	13	13	13	13	13	13	13	13
relocation-overlap	13	13	13	13	13	13	13	13	13	12
translocation	111	50	57	43	50	50	50	50	50	62
translocation-insertion	13	12	12	12	12	12	12	12	12	13
translocation-insertion_ATGC	13	13	13	13	13	13	13	13	13	13
translocation-inserted_gap	13	13	13	13	13	13	13	13	13	13
translocation-overlap	13	13	13	13	13	13	13	13	13	11
inversion	534	530	530	528	529	530	531	526	530	528
reshuffling	2585	2585	2585	2585	2585	2585	2585	2585	2585	2536
substitution	115	81	81	81	80	81	89	68	81	84
gap	49	46	46	46	46	46	48	46	45	34
inserted_gap	21	21	21	21	21	21	21	21	20	16
mapped_seq	13	10	11	6	10	10	10	10	10	10
unaligned_sequence	13	13	13	13	13	13	13	13	13	13

We did not expect NucDiff to be able to detect all simulated differences of most types. This is confirmed in the results presented in Table 1, where NucDiff misses many differences of several types, no matter what parameter settings were used. A small deviation from the simulated results was expected since the fixed 30 bp limit for lengths of duplications in reference and query sequences and relocated blocks is much lower than the variable NUCmer and delta-filter thresholds. Another reason for the result deviation is that some difference locations were shifted a few bp due to accidental base similarity at the region borders. In such cases, the differences were considered wrongly resolved in spite of correctly detected types. These reasons are applicable to all difference types with the observed deviation to a greater or lesser extent. All other reasons are related to the chosen NUCmer and delta-filter parameter settings and NucDiff limitations and are discussed below.

The detailed results from [Additional file 2] indicate that increasing the alignment extension distance (−b parameter) led to the loss of information about repeat related local differences and inverted, relocated and substituted fragments. With a greater -b parameter value, NUCmer more successfully expands low scoring regions. It enables detection of more differences inside fragments and a reduction of the number of aligned fragments. However, at the same time, it does not allow tracking of possible locations of query regions involved in differences in the reference sequences. This leads to loss of information about the repeated, inverted and substituted nature of the regions. Changing the maximal exact match length (−l parameter) did not influence significantly on the obtained results within the considered simulations. Increasing the parameter value for minimum alignment identity (−i parameter) (see columns l65 and QUAST-like in Table 1) led to an increased number of wrongly discarded valid mapped short fragments as well as query sequences containing even a small number of short and medium length differences.

Increasing the values for the minimum cluster length (−c parameter) increases the number of discarded correct query sequences and discarded valid mapped fragments. This leads to 1) the undesirable loss of information about the inverted, relocated and translocated nature of some fragments and 2) the misrepresentation of correct query sequences as being unaligned.

Additional result deviations can be explained by the specifics and limitations of the approach implemented in NucDiff independent on the parameter values used. First, due to some simplifications during the NucDiff structural difference detection step, NucDiff does not allow detection of both relocations/translocations and duplications at the same time in cases when simple relocations/translocations are followed by duplications (see [Additional file 1: Table S2], relocation case 2 and translocation case 1). In such cases, the differences are detected either as a combination of a simple relocation/translocation and a simple insertion or as a combination of a simple insertion and a duplication depending on the length of a relocated or translocated fragment.

Second, another problem with duplication detection occurs in situations when reference fragments are duplicated and inserted into query sequences somewhere far away from their original locations (see [Additional file 1: Table S2], insertions, case 2). The duplications are detected by NUCmer but are filtered out by the delta-filter program as being aligned fragments with smaller length*identity weighted LIS [longest increasing subset]. This option is set by the -q parameter and is always used in NucDiff. As a result, NucDiff detects such duplications as simple insertions.

Third, in cases with a combination of a gap and an inserted gap, the order of the gap and the inserted gap varies depending on whether a subsequence of N’s caused alignment fragmentation or not. Since in the simulated results a gap is always followed by an inserted gap, the number of correctly detected gaps was slightly lower than the expected number for all parameter settings. However, this behavior influences only the numbers in Table 1 but not the quality of the obtained results.

### Comparison with QUAST

Both NucDiff and QUAST use the NUCmer package in their pipeline. However, QUAST only provides information about the locations of regions where the reference sequences were split during the alignment process and specifies the general reasons for the alignment fragmentations (e.g. local misassembly, relocation and so on). As with NucDiff, we calculated the number of correctly detected simulated differences. Since QUAST only separates the differences into broad categories, it is not possible to make direct one-to-one comparisons. We therefore grouped the simulated differences into types as described in [Additional file 1: Table S5]. A simulated difference is considered correctly detected if it overlaps with a QUAST difference that belongs to the same general category. In cases with repeat related types, a difference is considered correctly detected when one of the repeated fragments involved in the simulated difference overlaps with the QUAST difference. The obtained average total number for each type of difference is shown in Table 1. The detailed results for each simulated case (see in [Additional file 1: Table S2]) can be found in the [Additional file 2].

As expected, the results presented in Table 1 show that QUAST, as well as NucDiff, was not able to detect all simulated differences in most groups. The small deviation of QUAST results in all problematic groups can also be explained by the introduced 30 bp limit for lengths of duplications in reference and query sequences and relocated blocks and shifted locations of some differences. However, there are some additional reasons specific to QUAST.

First, QUAST does not output any information about the locations of small differences obtained after parsing the results given by the show-snps package, only providing information about their total number. This is reflected in a large deviation between the numbers of simulated and detected insertions, deletions, substitutions, gaps, and inserted gaps. Second, QUAST is unable to distinguish differences of several types at the identical locations. For example, duplications and reshufflings were not reported as stand-alone differences when they were located together with relocations or translocations. The same is also true for insertions and deletions when they were introduced between inverted and reshuffled blocks. Third, the comparison of the QUAST results with the NucDiff results obtained with the QUAST-like parameters settings suggests that QUAST has its own internal length threshold for filtering mapped fragments. This value is somewhat higher than the NUCmer -c parameter value used. This led to a reduced number of correctly detected relocation and translocation events.

During comparison of the QUAST results with the NucDiff results obtained with the QUAST-like settings, we noticed that QUAST was able to detect more duplication and translocation events. This can be explained by less strict requirements for correspondence between the simulated and obtained types for QUAST. For example, in situations where NucDiff detected simple translocations and duplications as translocation with insertions and simple insertions, respectively (see translocation case 1 in [Aditional file 1: Table S2]), the differences were considered wrongly resolved by NucDiff and correctly resolved by QUAST. The same problem is also applicable to simple relocations. However, since fewer relocations were detected by QUAST because of its filtering approach, the significant divergence between numbers is not apparent in Table 1.

### Comparison with dnadiff

The NucDiff, dnadiff and QUAST tools provide a quantification of the differences between two sets of genomes. In this section, we compare the numbers output by these tools. Due to the way these tools report their results, it is very difficult to make a fair comparison between them. All tools were run on the same simulated genome described in Datasets section. NUCmer, whose output was used by NucDiff and dnadiff, was run with the QUAST-like parameter settings (see [Additional file 1: Table S4]). Since dnadiff only provides the number of differences and not their locations, we cannot know for sure whether the differences are actually in the same places as reported by the other tools. To perform the comparison, we created a set of categories suitable for comparison and grouped the differences reported into these categories (see [Additional file 1: Table S6] for grouping). The results are presented in Table 2.

**Table 2:** Number of simulated differences (Truth) and differences obtained by NucDiff, dnadiff and QUAST

Group	Truth	NucDiff	dnadiff	QUAST
nonTandem	3448	6717	7460	2814
Tandem	119	116	116	0
Substitutions	164	423	234	354
Relocations	2854	2802	2211	185
Translocations	163	137	137	117
Inversions	1068	1060	1060	1053
UnalignedSeq	13	21	16	21

In the nonTandem group, the values shown for the simulated differences (Truth) and QUAST are the number of events, while in the other columns the values are the sum of the number of bases involved in the differences for short and medium local differences (found by the show-snps program) and the number of events of long local differences (those causing alignment fragmentation). In the Inversions group, the numbers of simulated inversions and inversions found by NucDiff were multiplied by two to enable a fair comparison, because QUAST and dnadiff report the number of fragment ends, while NucDiff reports the number of fragments. In the Substitutions group, the values shown are the number of bases, while in the other rows the values are the number of events. The reshuffling differences are contained in the nonTandem group for QUAST, but placed in the Relocations group in all other cases

The results showed that the obtained counts for NucDiff and dnadiff are largely similar, while QUAST has a tendency to detect fewer differences than NucDiff and dnadiff in almost all categories. A large deviation between the results from QUAST and the other tools was observed in the nonTandem and Relocations groups. In both cases, it can be explained by how the comparison is performed and not necessarily by the performance of the tool.

### Comparison of several assemblies of the same read set to the same reference genome

**Fig. 5:**
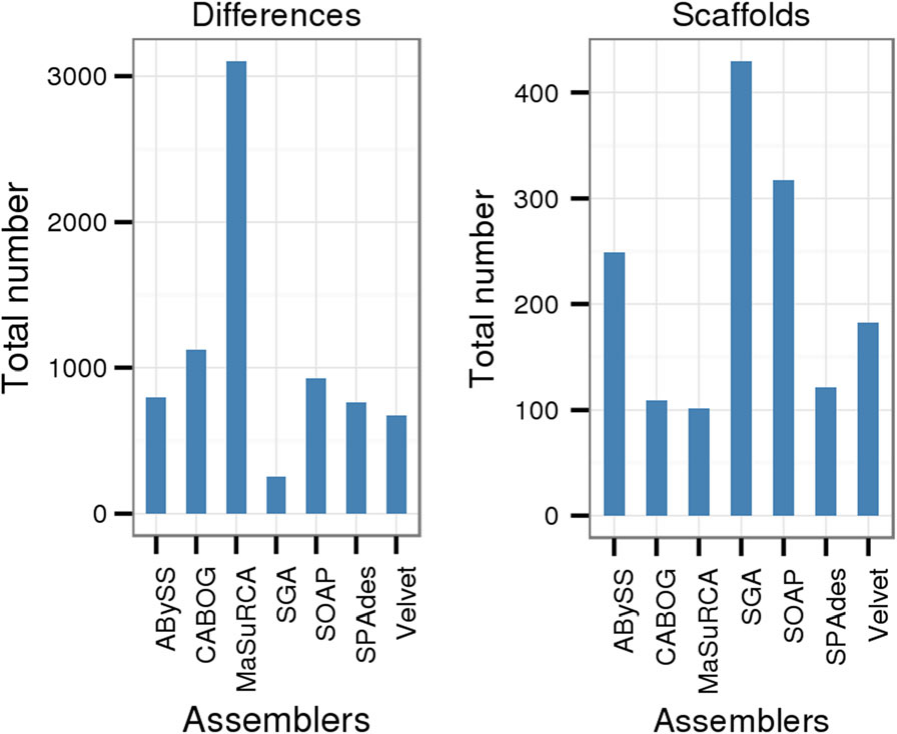
Total number of scaffolds and differences. The differences were found with the default NucDiff parameter settings for each assembly

We downloaded assemblies of the same *V. cholerae* read set as described in the Datasets section, and compared them to a *V. cholerae* reference using NucDiff with default parameter settings (see in [Additional file 1: Table S4]). The number of detected differences is presented in [Additional file 1: Figure S3]. The total number of scaffolds and differences is shown in Fig. 5. The resulting GFF3 files with mapped blocks and differences (shown with reference-based coordinates) were displayed using the IGV genome browser, and an example of assembly comparison is shown in [Additional file 1: Figure S4]. As is evident, we were able not only to compare quantitative metrics (i.e. the number of each type of difference, the number of uncovered reference bases, etc.) but also to analyse the placement of contigs/scaffolds and differences relative to each other and the exact location of the different types of detected differences.

Based on the obtained results, it is possible to conclude for the given examples that SGA gives the most fragmented assembly compared with other assemblers, while MaSuRCA gives the solution with the highest number of errors (differences are considered as errors in this case, since we are comparing to a good quality reference genome), mainly suffering from substitution errors (2839 out of 3106 differences). SOAPdenovo has rather high numbers of errors in all categories, confirming the result from GAGE-B using QUAST, which states that SOAPdenovo has “a larger number of errors than most other methods” [12]. It is also possible to see the large fragmentation in the SGA assembly and the large total number of differences in the MaSuRCA assembly by visualisation in IGV in [Additional file 1: Figure S4a and c].

According to GAGE-B results, MaSuRCA has produced the assembly with the best N50 size. However, in our experience, MaSuRCA did not distinguish itself when compared to other assemblers. All assemblers have managed to resolve some regions where most of the other assemblers failed to get continuous solutions. In addition, we noticed that there are some differences that were produced by all assemblers in the same places. For example, we detected two deletions, one of length 1255 bp, overlapping with two open reading frames of a transposase ([Additional file 1: Figure S5a]) and a second of length 1367 bp, overlapping with two genes of unknown function ([Additional file 1: Figure S5b]), and many short insertions, deletions and substitutions through the genome. We suspect that such errors may actually be true variations between the sequenced genome and the reference genome rather than errors in the assemblies in many cases. For example, in the case of the transposase, this may have inserted itself in the strain sequenced for the reference genome, while it was absent from the DNA of the strain sequenced for GAGE-B.

### Comparison of genomes from different strains of the same species

**Fig. 6:**
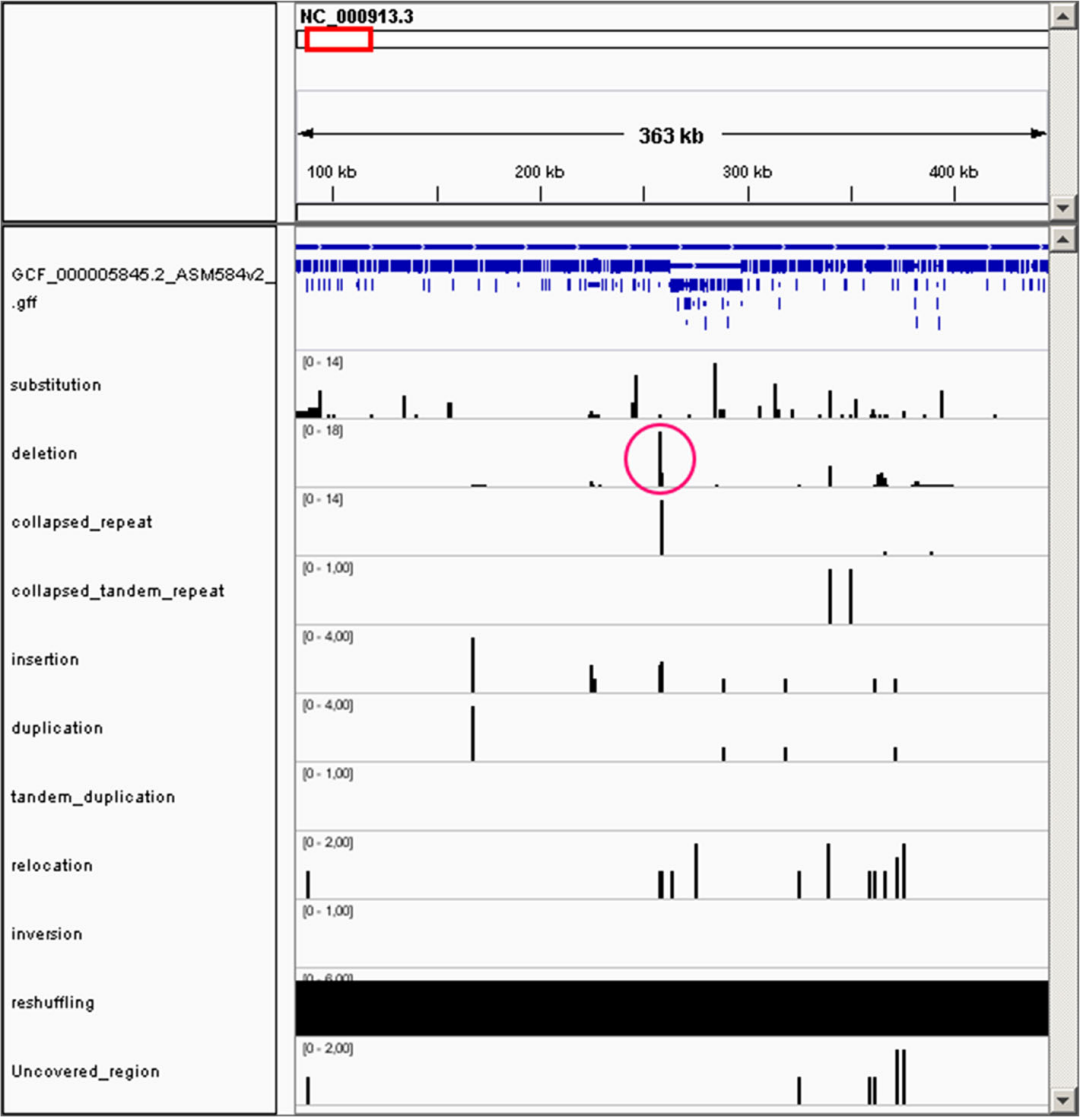
Comparison of 21 *E. coli* K12 genomes with *E. coli* K12 MG1655. The first entry corresponds to the *E. coli* K12 MG1655 annotation. All other entries correspond to the difference types. Vertical bars show the number of genomes having a difference of the specified type at the current position. Each difference type entry has its own scale and is adjusted depending on the maximum value presented in the displayed area. The encircled area corresponds to the deletion of bases (257908–258,674 in U00096.3), which is present in 15 out of 21 genomes. These bases correspond to a mobile element with coordinates 257,908–258,675. The current region of the reference chromosome, shown on the figure, was reshuffled in 6 genomes and is represented as one solid thick line in the reshuffling entry in the figure

With NucDiff, it is also possible to compare genomes of different strains of the same species to show genomic differences between them. We have compared the genomes of 21 different strains of *E. coli* K12 available in the NCBI database to the *E. coli* K12 MG1655 as the reference genome. We have calculated the total number of differences of each type at every base of each query reference. The result was saved in the bedGraph format and uploaded into the IGV genome browser together with the *E. coli* K12 MG1655 annotation (see Fig. 6).

The results show that the differences are not distributed randomly, they tend to be clustered in some locations. For example, in 15 out of 21 genomes there is a deletion of bases, starting from base 257,908 and ending with base 258,674 in U00096.3 (a circle in Fig. 6). These bases correspond to a mobile element with almost the same starting coordinate and ending in position 258,675.

## Discussion

In this paper, we have described a tool, called NucDiff, which detects and describes the differences between any two sets of closely related DNA sequences according to our comprehensive classification scheme. The tool has several properties that make it very useful for doing comparative analysis of assemblies and reference genomes. 1) It is able to work with very fragmented genome assemblies and genomes with various structural rearrangements. We have demonstrated this with the *V. cholerae* assemblies and *E. coli* K12 reference genomes. Moreover, NucDiff is in many cases able to detect differences that are associated with repeated regions (for example, in case of duplication, tandem duplication, collapsed repeat and tandem collapsed repeat differences). However, it is not able to detect such associations for simple insertions and simple deletions found between mapped blocks. 2) The tool gives information about the locations and types of differences. This information is stored in the widely used GFF3 format with both query-based and reference-based coordinates, which can be used with existing genome browsers for visualizing the differences. The NucDiff output also enables users to incorporate the tool in a large variety of applications where detecting differences is either the final goal or as a component in an analysis pipeline. 3) The tool enables visualisation of alignment results, as a result of outputting differences in the GFF3 format. We have shown two different applications of visualisation to further inspect the results. First, by uploading the files with locations of differences and mapped blocks for all compared assemblies at once into a genome browser. This approach enables comparison of all differences at any level of detail across all datasets to look for patterns. Second, by uploading the files with the summarized counts of all difference types in all query genomes for each reference sequence base. This visualisation gives a better overview of the comparison analysis results when the number of compared genomes is high, revealing common and distinct patterns in the structures of genome sequences. However, such an approach leads to the loss of some information about differences (e.g. which query genome(s) have the specific differences and the difference locations in these genome(s)).

The benchmarking results showed that the NucDiff output depends on the NUCmer and delta-filter parameters values. The values mainly influence the types of differences and not the total number, revealing or hiding information about the repeated, inverted, substituted, or relocated nature of the short- and medium-sized differences. The locations of regions containing differences remain the same in most cases. As for NucDiff result quality, we have noticed systematic loss of information about the repeated nature of some differences in specific cases. This was due to the limitations of the approach implemented in NucDiff.

Together with NUCmer, MUMmer provides another alignment program called PROmer. Unlike NUCmer, PROmer can be used for highly divergent sequences that show little DNA sequence conservation. Since both tools output the alignments results in the same format, it is possible to run NucDiff with PROmer output file as an input parameter, thus enabling detection of differences between two highly divergent sequences.

There are similarities between NucDiff and QUAST, a software tool for comparing assemblies to reference genomes. Both use NUCmer as a part of their analysis pipeline to align the input sequences. However, QUAST assesses genome quality mainly based on contiguity and gene complement completeness, producing various reports, plots and tables. QUAST will output quality metrics (e.g. number of misassemblies, indels and so on) only when a reference genome is available. In this case, it reports information about similar reference and query sequences, unmapped query sequences, and the locations of the regions where the reference and query sequences were split during the alignment process, giving general explanations for the fragmentation. It does not output the locations of small indels and substitutions obtained after parsing results given by the show-snps package. It provides only the raw show-snps output and summary statistics for these types of differences. Our experiments showed that QUAST tends to count several differences located at the same position as one difference. Comparing to QUAST, our tool is also able to give more detailed information about the locations of all differences as well as a more detailed classification of them. In addition, NucDiff allows the users to upload the results to different genome browsers, while QUAST output can be directly visualised only in its own genome browser, Icarus, that does not handle uploading of additional tracks.

We have also compared NucDiff with dnadiff. Both tools parse the NUCmer output and produce detailed information about the differences between two sets of sequences. Their results are very similar, but, in contrast to NucDiff, dnadiff does not allow visualization of differences and is not able to quantify them at the same level of detail.

Our results from analyses of different real assemblies have revealed a complication related to assembly comparison. It is not always enough to only use the quality and contiguity summary metrics when choosing the “best” assembly. The ability to visualize results and manually inspect the regions where the differences are located may dramatically influence this choice.

## Conclusions

We present the tool NucDiff for the comparison of two sets of closely related sequences. NucDiff outputs information about the types and locations of the differences between the sequences. Special attention has been paid to detection of differences involving repeated regions. All differences are categorized according to a proposed detailed classification scheme. The output from NucDiff enables the user to visualise the results using a genome browser, and we demonstrate two different applications of such visualisations. The ability to 1) give detailed information about the differences, 2) handle small local differences as well as structural rearrangements, and 3) visualise the comparison results makes NucDiff convenient for whole-genome sequence comparison or as an intermediate step in an analysis pipeline.

## Additional files

Additional file 1: **Figure S1.** Reference fragments placement order depending on query fragment orientations during detection of local differences. **Figure S2.** Circular genome alignment alternatives. **Figure S3.** Number of differences in each category obtained by NucDiff with the default parameter settings for all assemblers. **Figure S4.** Comparison of multiple assemblies against one reference using NucDiff. **Figure S5.** Examples of detection of long deletions located in all assemblies at the same place in the reference sequence. **Table S1.** Alignment fragmentation cases caused by simple differences. **Table S2.** Genome modifications implemented during the simulation process. **Table S3.** List of *E. coli* genomes used in the Comparison of genomes from different strains of the same species section. **Table S4.** Parameter values used for each parameter settings. **Table S5.** Correspondence between the QUAST difference types and the simulated difference types. **Table S6.** Correspondence between the QUAST, dnadiff and NucDiff difference types and the expected difference types. (PDF 989 kb)

Additional file 2: Detailed results for Table 1. (TXT 472 kb)

## Abbreviation

WGA: Whole-genome alignment
