# Supplementary material for: NucDiff: in-depth characterization and annotation of differences between two sets of DNA sequences
Source: BMC Bioinformatics. 2017 Jul 12;18:338. doi: 10.1186/s12859-017-1748-z (PMC5508607; doi:10.1186/s12859-017-1748-z)
Supplement: Supplementary file 1 — Figure S1. Reference fragments placement order depending on query fragment orientations during detection of local differences. Figure S2. Circular genome alignment alternatives. Figure S3. Number of differences in each category obtained by NucDiff with the default parameter settings for all assemblers. Figure S4. Comparison of multiple assemblies against one reference using NucDiff. Figure S5. Examples of detection of long deletions located in all assemblies at the same place in the reference sequence. Table S1. Alignment fragmentation cases caused by simple differences. Table S2. Genome modifications implemented during the simulation process. Table S3. List of E. coli genomes used in the Comparison of genomes from different strains of the same species section. Table S4. Parameter values used for each parameter settings. Table S5. Correspondence between the QUAST difference types and the simulated difference types. Table S6. Correspondence between the QUAST, dnadiff and NucDiff difference types and the expected difference types. (PDF 989 kb) [file 12859_2017_1748_MOESM1_ESM.pdf]

NucDiff: in-depth characterization and annotation of differences between two sets of DNA sequences:

Supplementary materials

Ksenia Khelik, Karin Lagesen, Geir Kjetil Sandve, Torbjørn Rognes, Alexander Johan Nederbragt

Supplementary results

Effect of different MUMmer parameters: result comparison approach

In general, the simulated difference is assumed to be correctly detected if its location intersects with the location of a NucDiff-detected difference of the same type. However, some exceptions had to be made in order to allow a fair comparison in cases where there are identical bases nearby just by incident. First, in the cases with all types of deletions and simple relocations and translocations, the detected difference may be located not more than 3 bases before or after the simulated difference. Second, some differences are allowed to have several corresponding types, i.e. simulated simple relocations and translocations may be detected by NucDiff as simple relocations and translocations or relocations and translocations with overlap. In spite of the chosen NUCmer and delta-filter parameter values, we defined that all repeat related differences are detected as non-repeat related if they are shorter than 30 bases. If some fragment was relocated to another place in the query sequence, we defined that it is detected as a simple insertion if it is shorter than 30 bases. These limits are tool independent and were introduced to avoid detection of random duplications and fragment relocations.

Supplementary figures and tables

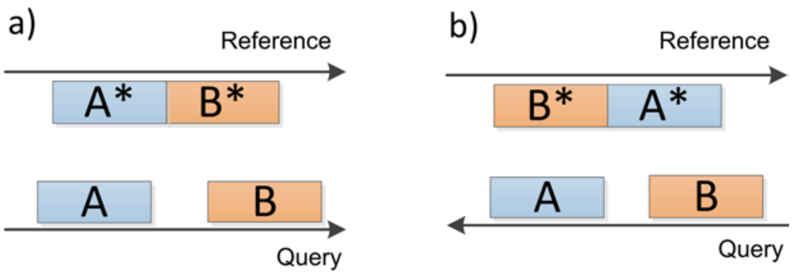

Figure S1 Reference fragments placement order depending on query fragment orientations during detection of local differences. a) case shows the placement order of A* and B* when A and B have the same orientation as A* and B*, b) case shows their placement order when A and B have the opposite orientation. The placement relation between A and B, A* and B* may be differ from what is shown here.

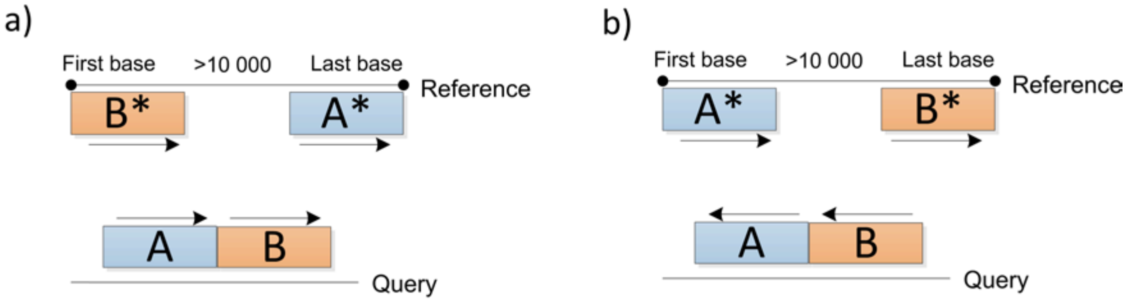

Figure S2 Circular genome alignment alternatives

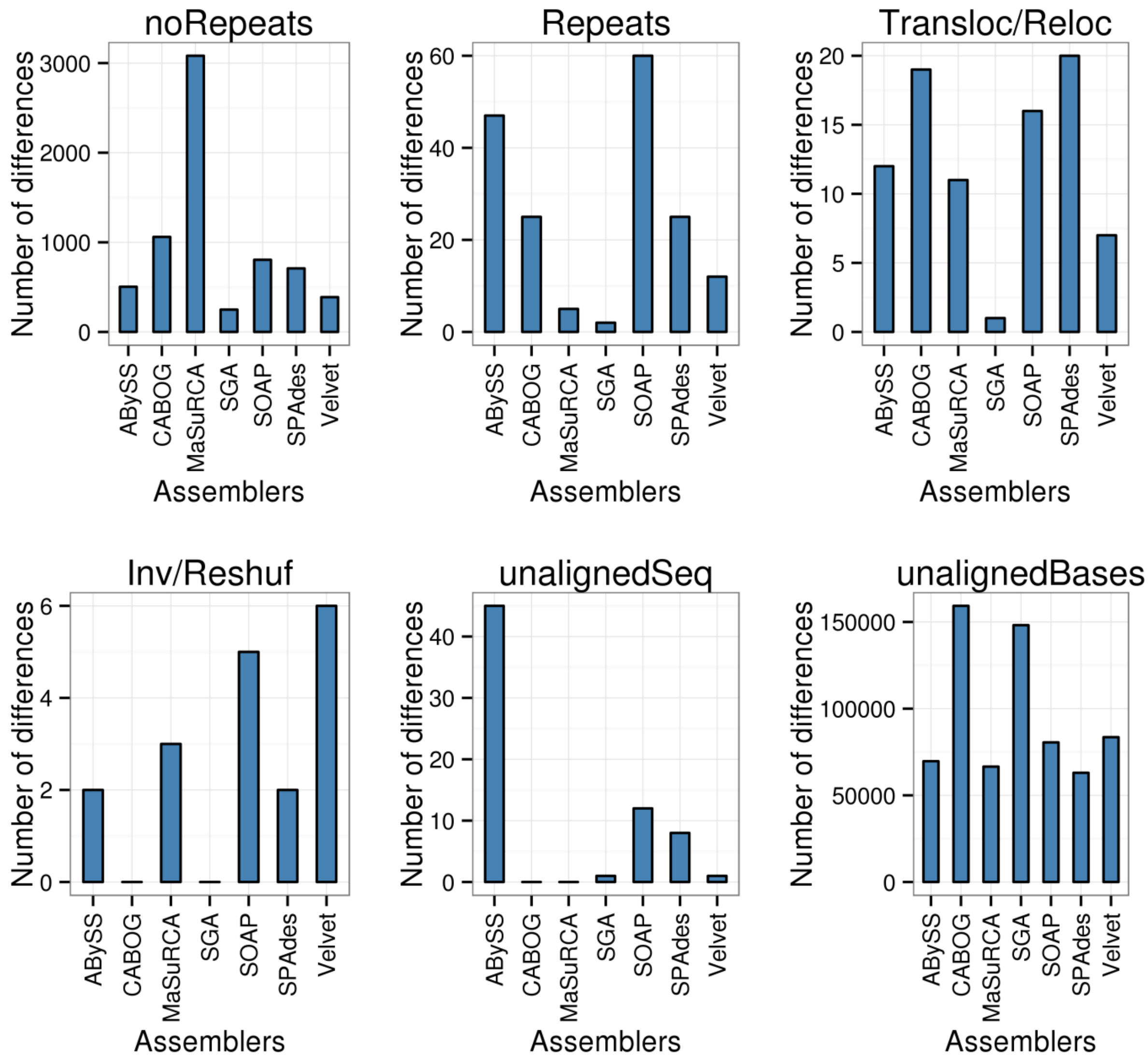

Figure S3 Number of differences in each category obtained by NucDiff with the default parameter setting for all assemblers. The noRepeats group consists of simple insertion, simple deletion, simple substitution, unaligned end and unaligned beginning differences. The Transloc/Reloc group consists of all types of translocations and relocations. Inv/Reshuf consists of inversions and reshufflings. The unalignedSeq group consists of unaligned sequence differences. The default parameter setting description can be found in Table S4.

a)

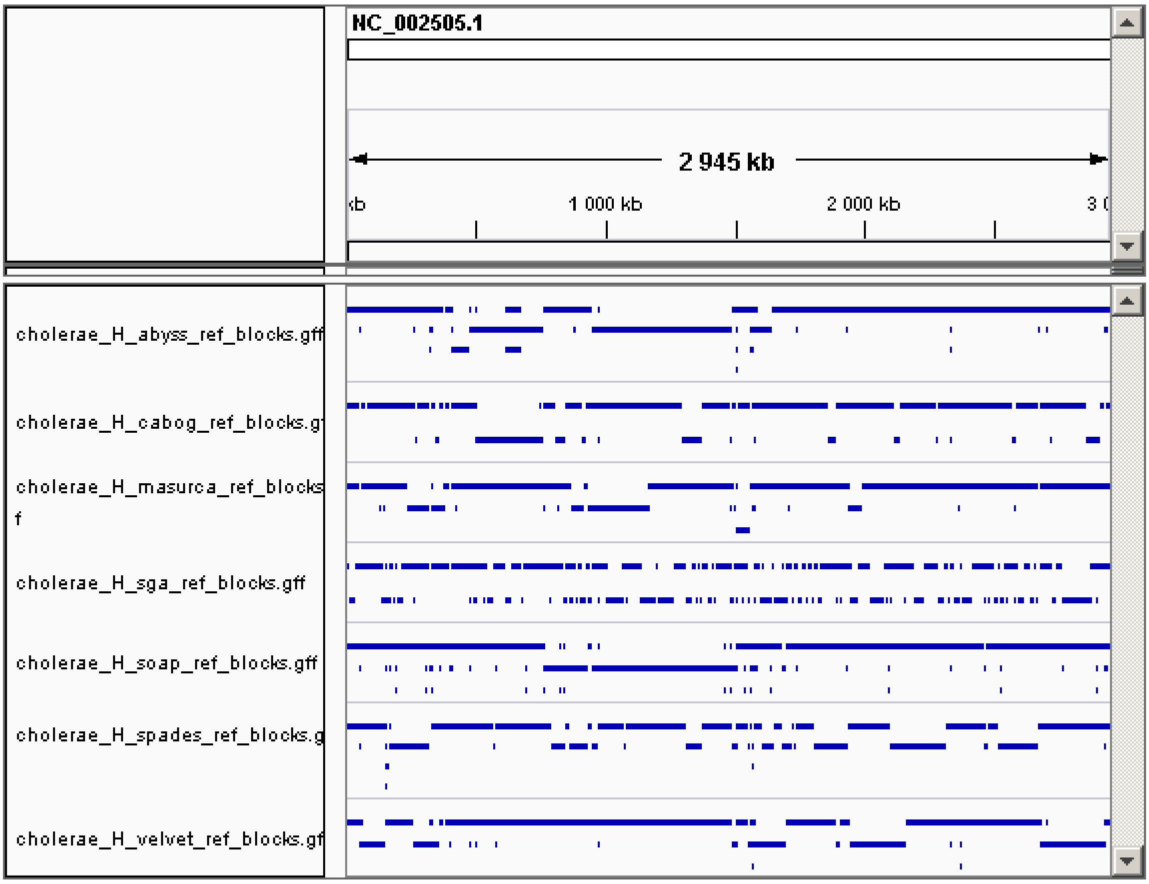
NC_002505.1
2 945 kb
1 000 kb
2 000 kb
cholerae_H_abyss_ref_blocks.gff
cholerae_H_cabog_ref_blocks.g
cholerae_H_masurca_ref_blocks
f
cholerae_H_sga_ref_blocks.gff
cholerae_H_soap_ref_blocks.gff
cholerae_H_spades_ref_blocks.g
cholerae_H_velvet_ref_blocks.gf

b)

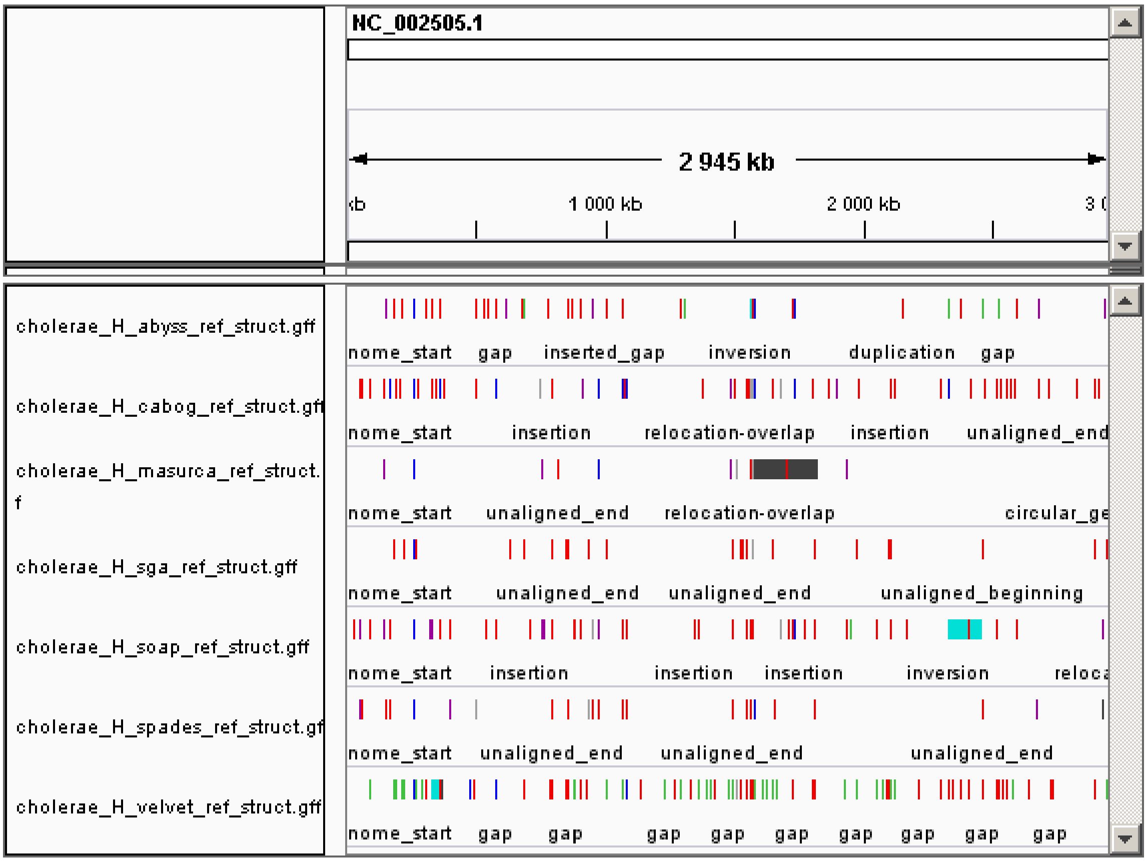
NC_002505.1
2 945 kb
1 000 kb
2 000 kb
cholerae_H_abyss_ref_struct.gff
nome_start gap inserted_gap inversion duplication gap
cholerae_H_cabog_ref_struct.gff
nome_start insertion relocation-overlap insertion unaligned_end
cholerae_H_masurca_ref_struct.
f
nome_start unaligned_end relocation-overlap
cholerae_H_sga_ref_struct.gff
nome_start unaligned_end unaligned_end unaligned_beginning
cholerae_H_soap_ref_struct.gff
nome_start insertion insertion insertion inversion
cholerae_H_spades_ref_struct.gff
nome_start unaligned_end unaligned_end unaligned_end
cholerae_H_velvet_ref_struct.gff
nome_start gap gap gap gap gap gap gap gap gap

c)

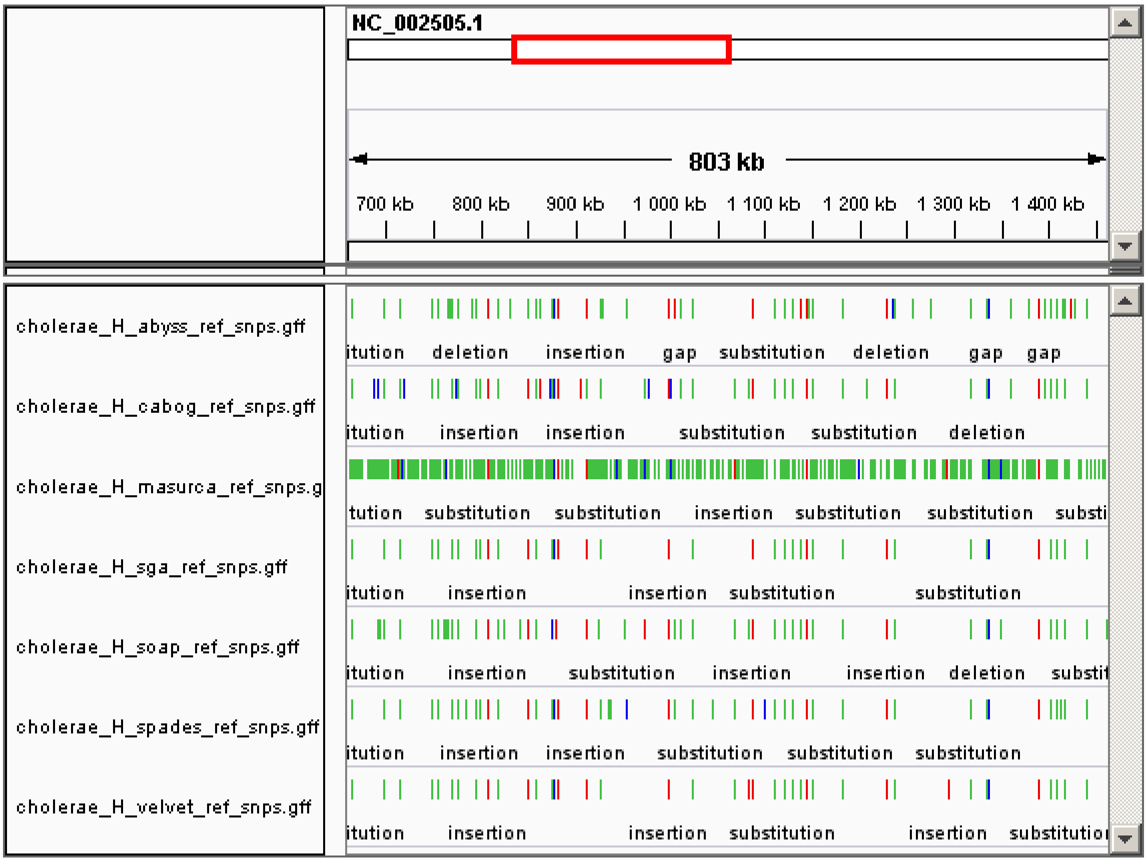

Figure S4 Comparison of multiple assemblies against one reference using NucDiff. The seven assemblies downloaded for the *V. cholerae* genome are created by the ABySS, CABOG, MaSuRCA, SGA, SOAPdenovo, SPAdes and Velvet assemblers based on a HiSeq read dataset. In a)-c) the top panel shows the focus area on the reference chromosome. The next seven panels corresponds to the reference mapped blocks in a), to the structural and long differences in b) and to the short and medium local differences in c).

a)

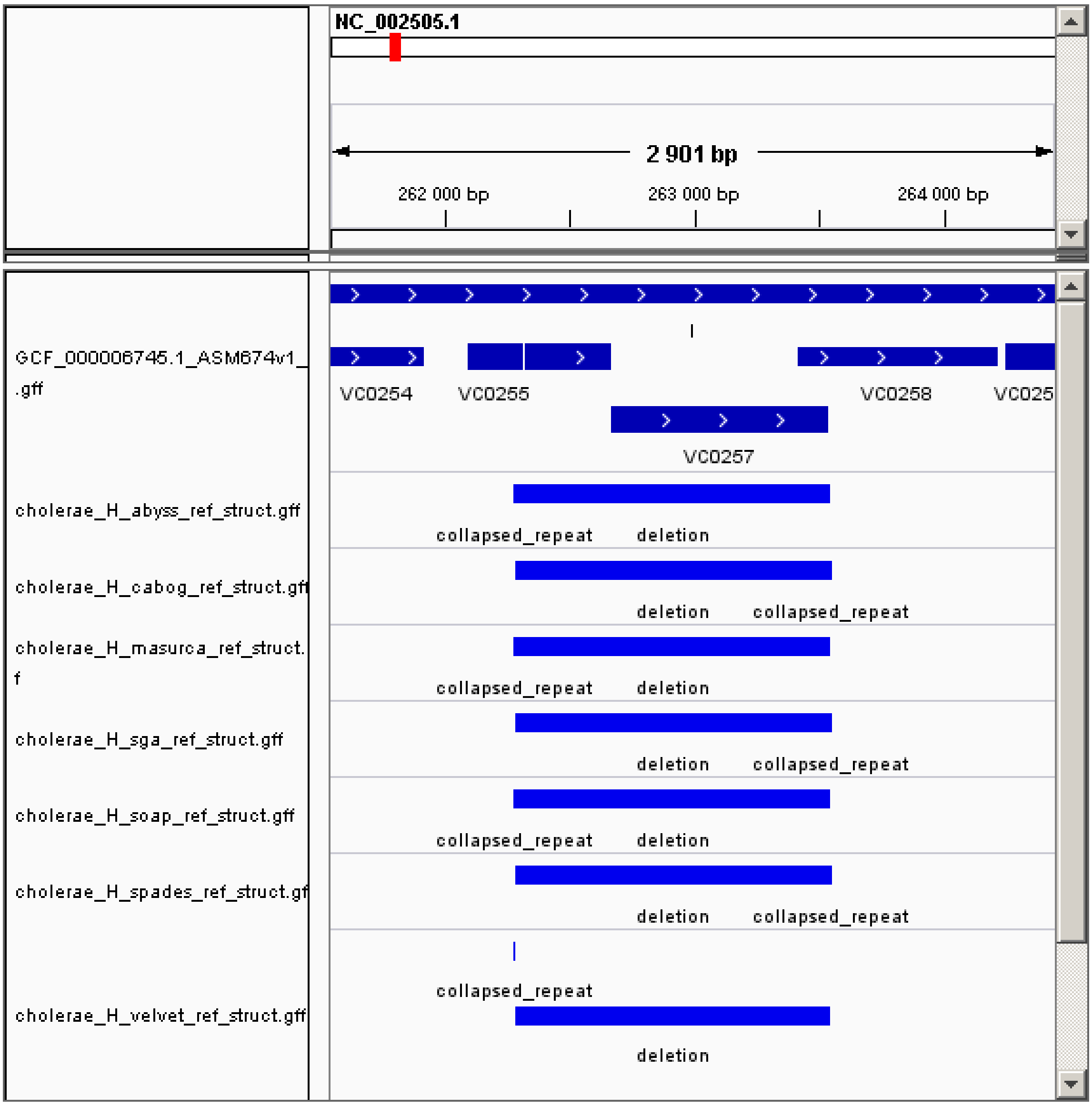
NC_002505.1
2 901 bp
262 000 bp
263 000 bp
264 000 bp
GCF_000006745.1_ASM674v1_.gff
VC0254
VC0255
VC0258
VC025
VC0257
cholerae_H_abyss_ref_struct.gff
collapsed_repeat
deletion
cholerae_H_cabog_ref_struct.gff
deletion
collapsed_repeat
cholerae_H_masurca_ref_struct.f
collapsed_repeat
deletion
cholerae_H_sga_ref_struct.gff
deletion
collapsed_repeat
cholerae_H_soap_ref_struct.gff
collapsed_repeat
deletion
cholerae_H_spades_ref_struct.gf
deletion
collapsed_repeat
cholerae_H_velvet_ref_struct.gff
collapsed_repeat
deletion

b)

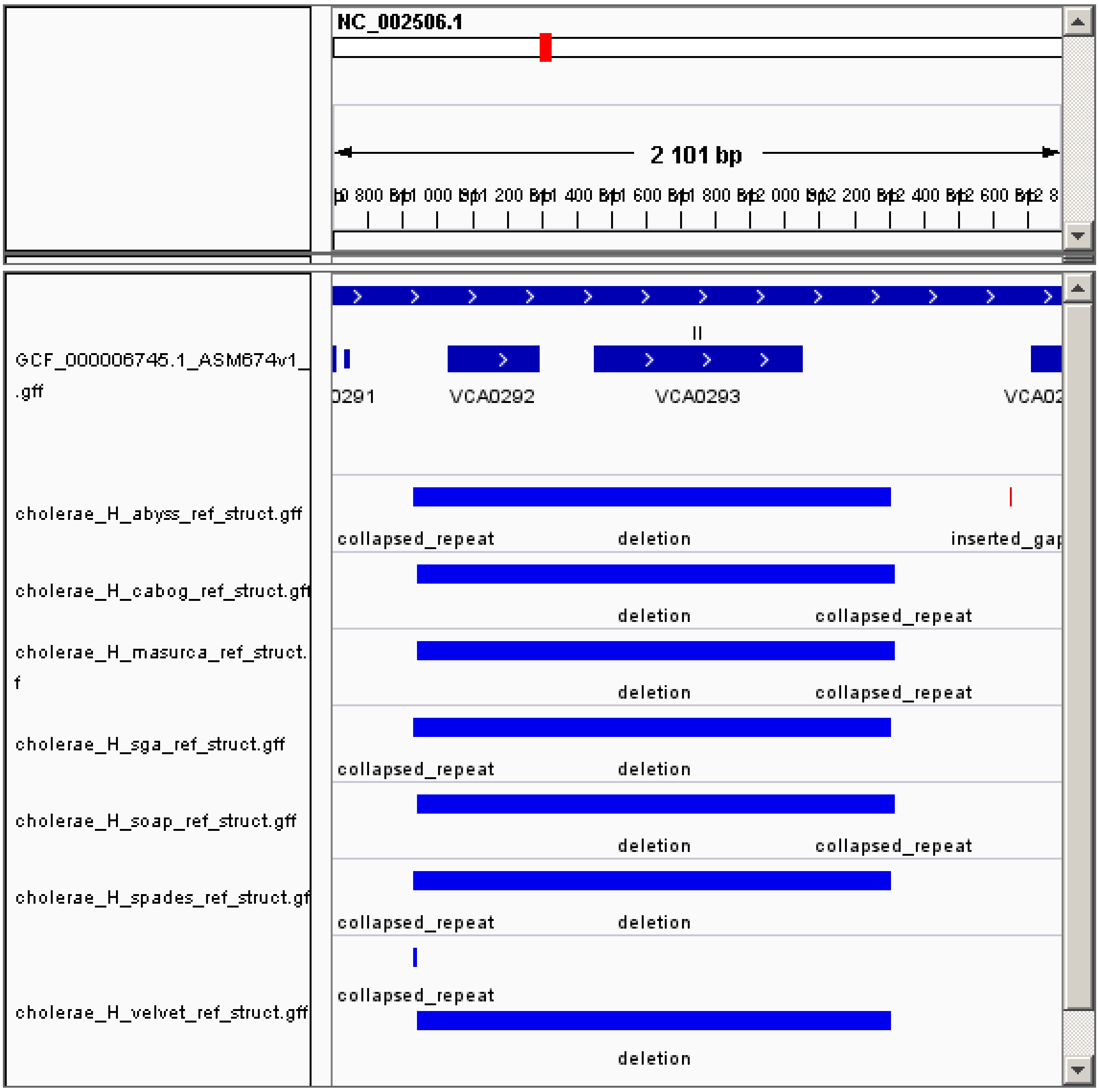

Figure S5 Examples of detection of long deletions located in all assemblies at the same place in the reference sequence. The deletion differences were found by NucDiff with the default parameter settings. The first entry corresponds to the *V. cholerae* annotation. In a) the deletion location coordinates are 262291 - 263545 bases in NC_002505.1. In b) the deletion location coordinates are 310944 - 312310 bases in NC_002506.1

Table S1 Alignment fragmentation cases caused by simple differences. K', M', R', T' are the reverse compliments of K, M, R, T, respectively. Len means length

Fragmentation case	Sequences	Differences	Symmetric fragmentation case
1. 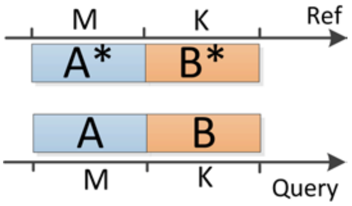	R: MK<br>Q: MK	no differences between fragments	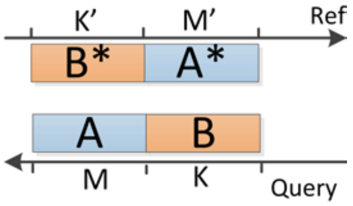
2. 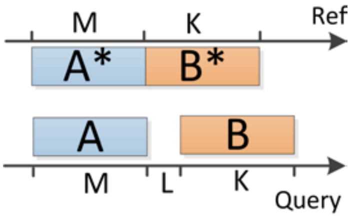	R: MK<br>Q: MLK	1) simple insertion<br>or<br>2) inserted gap	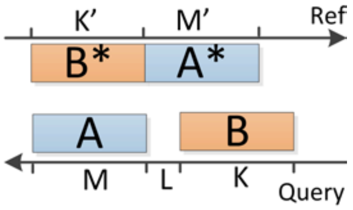
3. 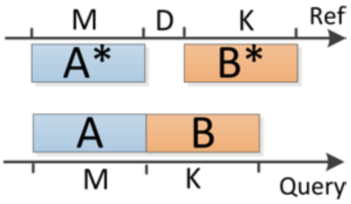	R: MDK<br>Q: MK	simple deletion	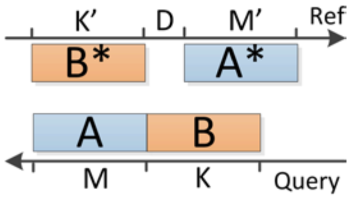
4.1 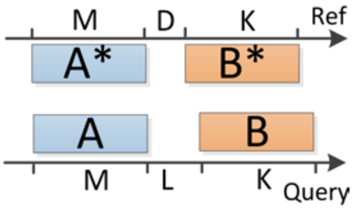	R: MDK<br>Q: MLK<br>Len(D)≤Len(L)	1) simple substitution and simple insertion<br>or<br>2) gap and inserted gap	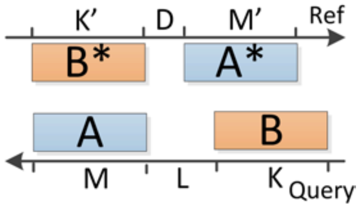

4.2<br>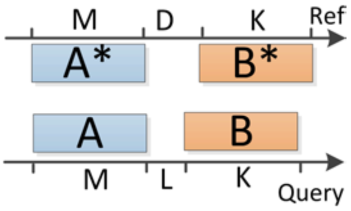	R: MDK<br>Q: MLK<br>Len(D)>Len(L)	1) simple substitution and simple deletion<br>or<br>2) gap and simple deletion	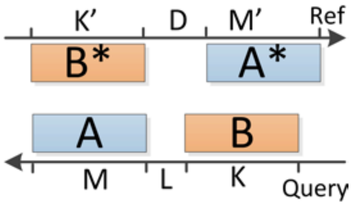
5.<br>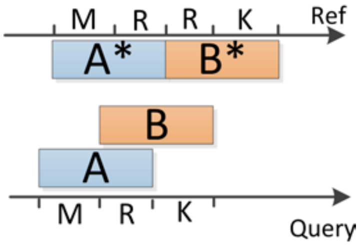	R: MRRK<br>Q: MRK	collapsed tandem repeat	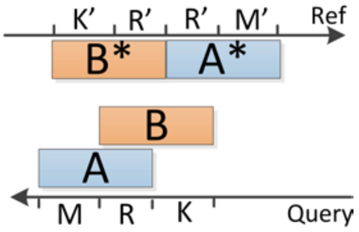
6.<br>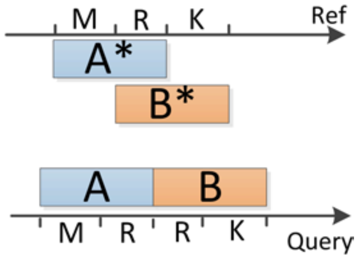	R: MRK<br>Q: MRRK	tandem duplication	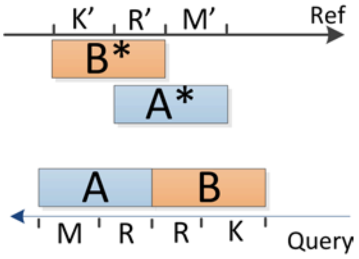
7.<br>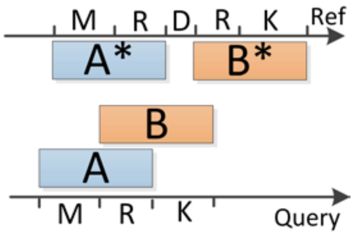	R: MRDRK<br>Q: MRK	simple deletion and collapsed repeat	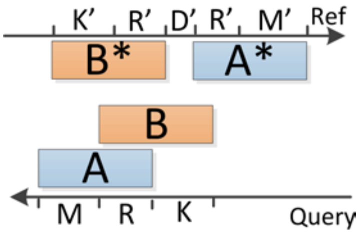

<table>
<tr>
<td>8.
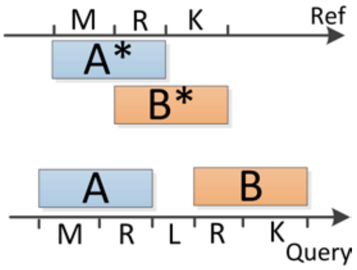
</td>
<td>R: MRK
Q: MRLRK</td>
<td>simple insertion

and duplication</td>
<td>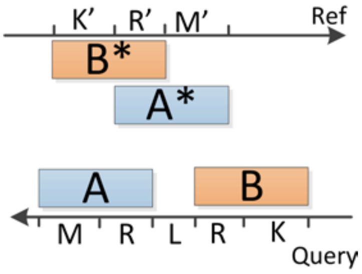
</td>
</tr>
<tr>
<td>9.1
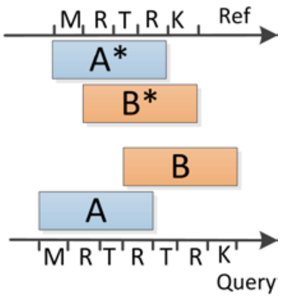
</td>
<td>R: MRTRK
Q: MRTRTRK</td>
<td>tandem duplication</td>
<td>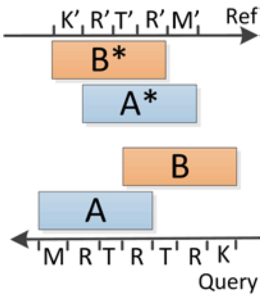
</td>
</tr>
<tr>
<td>9.2
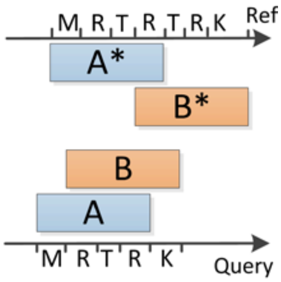
</td>
<td>R: MRTRTRK

Q: MRTRK</td>
<td>collapsed tandem repeat</td>
<td>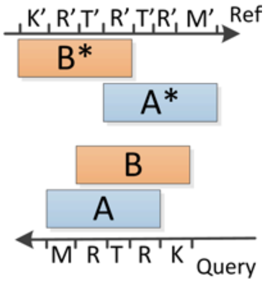
</td>
</tr>
</table>

In Table S1 case 1 may appear only after merging the fragments in the nested fragment cases. It will never be met in the NUCmer output. In all cases with overlaps between query or reference fragments (cases 5, 6, 7, and 8), the lengths of corresponding differences (L_{diff}) are calculated based on the length of the overlap and information about the differences inside fragment by the following formulas:

a) If there is an overlap between reference fragments (cases 6 and 8):

$L_{diff} = L_{ref} + L_{ins} - L_{del}$, where

L_{ref} - the length of the reference overlapping region

L_{ins} - the length of an insertion that have a corresponding reference coordinate within the overlapping region

L_{del} - the length of a deletion that have a corresponding reference coordinate within the overlapping region

b) If there is an overlap between query fragments (cases 5 and 7):

$L_{diff} = L_q - L_{ins} + L_{del}$, where

L_q - the length of the query overlapping region

L_{ins} - the length of an insertion that locates within the overlapping region

L_{del} - the length of a deletion that locates within the overlapping region

In cases where there are overlaps between reference and query fragments (cases 9.1 and 9.2), the type and length of difference are determined based on the lengths of both overlaps and information about the differences inside fragments. First, NucDiff calculates the difference length (L_{diff}) by the formula:

$L_{diff} = L_q - L_{ref} + L_{ins} + L_{del}$, where

L_{diff} - the length of overlap between reference fragments

L_{ref} - the length of overlap between query fragments

L_{ins} - the length of all simple insertions and inserted gaps within the query overlapping region

L_{del} - the length of all deletions within the query overlapping region

Then it determines the placement case and corresponding difference type:

If $L_{diff}>0$, then there is a case 9.2

If $L_{diff}<0$, then there is a case 9.1

If $L_{diff}=0$, then there is no differences between fragments

Table S2 Genome modifications implemented during the simulation process. R and Q denote reference and query genomes respectively. Letters A, B, C,K ,x, y denote reference and query sequence regions. Diff means difference. A’, B’ and F’ are the reverse complements of A,B and F, respectively.

**Deletions**	**Insertions**
1.R: ABC<br>Q: AC<br>Diff: deletion<br><br>2. R: ABCxBy<br>Q: AC<br>Diff: deletion<br><br>3. R: ABCxBy<br>Q: ACxBy<br>Diff: collapsed repeat<br><br>4. R: ABKBC<br>Q: ABC<br>Diff: deletion + collapsed repeat<br><br>5. R: ABBC<br>Q: ABC<br>Diff: tandem collapsed repeat<br><br>6. R: ABKBKBC<br>Q: ABKBC<br>Diff: tandem collapsed repeat	1. R: AC<br>Q: ABC<br>Diff: insertion<br><br>2. R: ACxBy<br>Q: ABCxBy<br>Diff: duplication<br><br>3. R: ABC<br>Q: ABKBC<br>Diff: insertion + duplication<br><br>4. R: ABCxKy<br>Q: ABKBCxKy<br>Diff: duplication + duplication<br><br>5. R:ABC<br>Q:ABBC<br>Diff: tandem duplication<br><br>6. R: ABKBC<br>Q: ABKBKBC<br>Diff: tandem duplication
**Substitutions**	**Gaps**
1. R: ABC<br>Q: AKC<br>Diff: substitutions (with and without insertions and deletions)<br><br>2. R: ABCxKy<br>Q: AKCxKy<br>Diff: substitution	1. R: ABC<br>Q: ANC<br>Diff: gap
**Relocations**	**Translocations**
1. R: ACxBy<br>Q: ABC<br>Diff: relocation<br><br>2. R: ABCxKy<br>Q: ABKBC<br>Diff: relocation + duplication<br><br>3. R: ABCxKy<br>Q: AKC<br>Diff: relocation<br><br>4. R:AxC<br>Q:AC<br>Diff: relocation<br><br>5a. R:AxC<br>Q:ABC (B consists of ATGC’s)<br>Diff: relocation with insertion	1. R1: ABC R2:K<br>Q: ABKBC<br>Diff:translocation + duplication<br><br>2. R1:A R2:C<br>Q:AC<br>Diff:translocation<br><br>3a. R1:A R2:C<br>Q:ABC (B consists of ATGC’s)<br>Diff: translocation with insertion<br><br>3b. R1:A R2:C<br>Q:ABC (B consists of N’s)<br>Diff: translocation with inserted gap<br><br>3c. R1:A R2:C<br>Q:ABC (B consists of ATGNC’s)<br>Diff: translocation with insertion and inserted gap

5b. R:AxC<br>Q:ABC (B consists of N's)<br>Diff: relocation with inserted gap<br>5c. R:AxC<br>Q:ABC (B consists of ATGNC's)<br>Diff: relocation with insertion and inserted gap<br><br>6. R:ABxBC<br>Q:ABC<br>Diff: relocation with overlap	4. R1:AB R2:BC<br>Q:ABC<br>Diff: translocation with overlap
**Inversions**	**Reshufflings**
1. R: ABC<br>Q: AB'C<br>Diff: inversion<br><br>2. R: AKBLC<br>Q: ATB'MC<br>Diff: inversions with and without insertions and deletions	1. R: ABLCKMD<br>Q: CTADWBK<br>Diff: reshufflings
**Additional tests**	**Unaligned sequences**
1. Combination of structural differences<br><br>R1: ABCDFxKy R2:L<br>Q: CA'DBF'KL<br>Diff: translocation, relocation, reshuffling and inversion events<br><br>2. Near similar repeats<br><br>R:AB1DB2C<br>Q: AB3C<br>Diff: small substitutions	1. Correct small query sequences<br><br>2. Completely wrong query sequences

In the simulated modifications the following lengths of regions were used:

1. Len(A/C) = 500 bases in all described cases, except reshuffling case
2. Len(x/y) =10500 bases in all described cases
3. Distance between each manipulation case = 10500
4. Len(B) = {5,20,50,65,85,88,100,150,200,250,300,350,400} bases in deletion (1,2,3,5), insertion (1,2,5) , relocation (1,5,6), translocation (2,3) and inversion (1) cases
5. Len(B/K) = {5,30,80,150,250,350,400} bases in deletion (4,6), insertion (3,4,6) , all substitution and relocation (2,3) cases
6. Len(B/N) = {5,30,80,150,250,350,400} bases in gap related cases
7. Len(B) = 250 and Len(K/L/T/M) = {0,5,250,400} bases in inversion (2) case
8. Len(A/B/C/D/K) = 250 and Len(L/M/T/W) = {0,5,250,400} bases in reshuffling case
9. Len(D/B/F/K/L) = 500 bases in additional test (1) case
10. Len(B1/B2/B3) = 150 and Len(D) = 45 bases in additional test (2) case. B3 contains one nucleotide difference with each of B1 and B2. The differences are located in the reference sequence at the same positions where B1 and B2 have two differences of one nucleotide lengths.
11. Len(correct/completely wrong seq) = {5,20,50,65,85,88,100,150,200,250,300,350,400} bases in all unaligned sequence cases.

Table S3 List of *E. coli* genomes used in the Comparison of genomes from different strains of the same species section.

Strain	Accession number	Strain	Accession number
AG100	NZ_LN832404.1	ER3454	NZ_CP010438.1
BW25113		ER3466	NZ_CP010442.1
BW2952	NC_012759.1	ER3475	NZ_CP010444.1
DH10B	NC_010473.1	ER3476	NZ_CP010440.1
EcHMS174Chr	NZ_LM993812.1	GM4792 Lac+	NZ_CP011342.2
EcRV308Chr			
ER3413	NZ_CP009789.1	MDS42	NC_020518.1
ER3435	NZ_CP010445.1	MG1655	U00096.3
	NZ_CP010439.1	MG1655_TMP32XR1	NZ_CP011343.2
ER3445	NZ_CP010441.1	MG1655_TMP32XR2	
ER3446	NZ_CP010443.1	W3110	NC_007779.1

Table S4 Parameter values used for each parameter settings. The default set shows NucDiff default values for NUCmer and delta-filter options. The Quast-like set describes parameter values as used in QUAST. Values in bold are different from the default ones.

Configuration sets	Parameter values	
default	--nucmer_opt "-l 20 -c 65 -b 200"	--filter_opt " -i 0"
c30	--nucmer_opt "-l 20 -c **30** -b 200"	--filter_opt " -i 0"
c120	--nucmer_opt "-l 20 -c **120** -b 200"	--filter_opt " -i 0"
l10	--nucmer_opt "-l **10** -c 65 -b 200"	--filter_opt " -i 0"
l65	--nucmer_opt "-l **65** -c 65 -b 200"	--filter_opt " -i 0"
b80	--nucmer_opt "-l 20 -c 65 -b **80**"	--filter_opt " -i 0"
b350	--nucmer_opt "-l 20 -c 65 -b **350**"	--filter_opt " -i 0"
quast-like	--nucmer_opt "-l **65** -c 65 -b 200"	--filter_opt " -i **95**"

-l - Minimum length of a maximal exact match

-c - Minimum cluster length

-b - Distance NUCmer will attempt to extend poor scoring regions before giving up

-i - Minimum alignment identity

All other NUCmer parameters, except --maxmatch, have the NUCmer default values and remained fixed in our tests. The --maxmatch parameter, which tells NUCmer to use all

anchor matches regardless of their uniqueness, is not used by default in NUCmer, but is required for NucDiff and thus is used in all tests.

As for the delta-filter filtering parameters, -q parameter (query alignment using length*identity weighted LIS [longest increasing subset]) is required for NucDiff to get the output results needed for the analysis and is present in all tests performed.

In the QUAST-like tests, we ran a test with the same parameter values used by QUAST, except for the -q parameter. It is not used by QUAST, but is required for NucDiff.

Table S5 Correspondence between the QUAST difference types and the simulated difference types

Simulated difference types	Qu difference types
insertion, deletion, substitution, duplication, tandem duplication, collapsed repeat, collapsed tandem repeat	local misassembly, indel
inversion	inversion
gap, inserted gap	fake: scaffold gap size wrong estimation (denoted as gap in Table 1 and Table 3)
relocation, relocation with overlap, relocation with insertion, relocation with insertion and inserted gap	relocation
relocation with inserted gap	relocation, fake: scaffold gap size wrong estimation
unaligned sequence	unaligned
all translocation types	translocation
reshuffling	local misassembly

Table S6 Correspondence between the QUAST, dnadiff, NucDiff difference types and the expected difference types

**Category**	**Expected/ NucDiff**	**dnadiff**	**QUAST**
nonTandem	insertion, deletion, substitution (from query_struct.gff), wrong beginning, wrong end, inserted gap, duplication, collapsed repeat	Insertions, TotalIndels	Local misassemblies, indels
Tandem	tandem duplication, collapsed tandem repeat	TandemIns	
Substitutions	substitution (from query_snps.gff), gap	TotalSNPs	mismatches
Relocations	All types of relocation, reshuffling	Relocations	Relocations, scaffold gap size misassemblies
Translocations	All types of translocations	Translocations	translocations
Inversions	inversion	Inversions	inversions
UnalignedSeq	Unaligned sequence	UnalignedSeqs	fully unaligned contigs
